# Ascorbic acid (vitamin C) synergistically enhances the therapeutic effect of targeted therapy in chronic lymphocytic leukemia

**DOI:** 10.1186/s13046-020-01738-0

**Published:** 2020-10-28

**Authors:** Walaa Darwiche, Cathy Gomila, Hakim Ouled-Haddou, Marie Naudot, Cécile Doualle, Pierre Morel, Florence Nguyen-Khac, Loïc Garçon, Jean-Pierre Marolleau, Hussein Ghamlouch

**Affiliations:** 1 EA 4666, HEMATIM, Université de Picardie Jules Verne, D408, 80054 Amiens Cedex, France; 2 Service d’Hématologie Clinique, Centre Hospitalier Universitaire Amiens Picardie, D408, 80054 Amiens Cedex, France; 3 EA 7516, CHIMERE, Université de Picardie Jules Verne, Amiens, France; 4 INSERM U1138, Centre de Recherche des Cordeliers, Sorbonne Université, Paris, France; 5 Hôpital Pitié-Salpêtrière, Sorbonne Université, APHP, Service d’Hématologie Biologique, Paris, France; 6 Service d’hématologie Biologique, Centre Hospitalier Universitaire Amiens Picardie, Amiens, France; 7 INSERM U1170, équipe labélisée Ligue Nationale Contre le Cancer, Gustave Roussy, 39 rue Camille Desmoulins, 94805 Villejuif Cedex, France

**Keywords:** Chronic lymphocytic leukemia, Ascorbic acid, Vitamin C, Cytotoxicity, Drug combination

## Abstract

**Background:**

Novel, less toxic, cost-effective and safe therapeutic strategies are needed to improve treatment of chronic lymphocytic leukemia (CLL). Ascorbic acid (AA, vitamin C) has shown a potential anti-cancer therapeutic activity in several cancers. However, the anti-cancer effects of ascorbic acid on CLL B-cells have not been extensively studied. We aimed in this study to evaluate the in vitro therapeutic activity using clinically relevant conditions.

**Methods:**

Primary CLL B-cells and two CLL cell lines were exposed to a dose that is clinically achievable by AA oral administration (250 μM), and cell death and potential mechanisms were assessed. The role of the protective CLL microenvironment was studied. Synergistic interaction between AA and CLL approved drugs (Ibrutinib, Idelalisib and Venetoclax) was also evaluated.

**Results:**

Ascorbic acid is cytotoxic for CLL B-cells at low dose (250 μM) but spares healthy B-cells. Ascorbic-acid-induced cytotoxicity involved pro-oxidant damage through the generation of reactive oxygen species in the extracellular media and in CLL cells, and induced caspase-dependent apoptosis. We also found that AA treatment overcame the supportive survival effect provided by microenvironment including bone marrow mesenchymal stem cells, T-cell cues (CD40L + IL-4), cytokines and hypoxia. Our data suggest that resistance to AA could be mediated by the expression of the enzyme catalase in some CLL samples and by the glucose metabolite pyruvate. We also demonstrated that AA synergistically potentiates the cytotoxicity of targeted therapies used in or being developed for CLL.

**Conclusion:**

These preclinical results point to AA as an adjuvant therapy with potential to further improve CLL treatments in combination with targeted therapies.

**Supplementary information:**

**Supplementary information** accompanies this paper at 10.1186/s13046-020-01738-0.

## Background

In the Western world, B-cell chronic lymphocytic leukemia (CLL) is the most common adult leukemia. It is characterized by the accumulation of CD5+ B lymphocytes in the blood, bone marrow, and secondary lymphoid tissues [1]. Until recently, the first-line therapy proposed to all patients was a combination of fludarabine-cyclophosphamide with CD20-specific immunochemotherapy (the antibody rituximab). The recent introduction of drugs targeting B-cell receptor signaling (such as the BTK inhibitor ibrutinib, the PI3K inhibitor idelalisib, and the BCL2 inhibitor venetoclax) has improved patient outcomes [2]. However, these drugs are rendered less effective over time by the emergence of resistance through (i) acquired somatic mutations in the genes coding for BTK, PLCG2 and BCL2, (ii) increased expression of anti-apoptotic genes (*BCL2*, *BCL2L1*, *MCL1*, etc.), and (iii) the CLL cells’ interaction with microenvironment [2]. Accordingly, there is still a need for novel, less toxic, cost-effective and safe treatment strategies for CLL.

High-dose vitamin C (i.e. L-ascorbic acid (AA)) was suggested as a potential anticancer agent for the first time in the 1970s by Pauling and Cameron [3]. More recently, preclinical data have confirmed the anticancer efficacy of AA and its selective cytotoxicity in different human cancers, both in vitro and in vivo [4–6]. Although several in vitro preclinical studies have shown that pharmacologically achievable concentrations of ascorbate have cytotoxic effects on cancer cells [4–6], AA has shown limited efficacy in some clinical trials [7, 8] but good efficacy in others [9]. In hematological malignancies, several studies have shown that AA is toxic for leukemic cells [10, 11] but does less damage to healthy cells [12, 13]. The major mechanism underlying AA’s anticancer activity is pro-oxidant damage through auto-oxidation. This leads to the generation of cytotoxic hydrogen peroxide (H_2_O_2_, i.e. a reactive oxygen species (ROS)) [5, 12, 14]. High concentrations of ROS are cytotoxic, via damage to DNA and mitochondria and the activation of apoptotic pathways [15].

The use of intravenous or oral administration route for AA has led to the impression that the compound’s anticancer effect is “controversial” because the route affects the maximum achievable concentration in plasma [16]. Earlier studies suggested that AA is cytotoxic at millimolar concentrations, which are achievable by intravenous injection but not by oral administration [4, 16]. However, revised interpretations and new knowledge about the pharmacokinetic properties of AA showed that a large oral dose can result in plasma concentrations of around 200 μM [4, 16–18]. Furthermore, several pharmaceutical formulations of vitamin C (e.g. liposomal encapsulations) can achieve levels of up to 400 μM [18, 19]. These new pharmacokinetic data and the results of previous clinical studies of orally administered vitamin C [3, 20] prompted us to investigate the in vitro effect of orally achievable concentrations of AA on CLL B-cells.

In CLL, little is known about the molecular mechanisms by which AA induces cytotoxicity, its interaction with the CLL microenvironment, and AA’s influence on the effectiveness of chemotherapy and targeted therapies. The few preclinical studies to have investigated AA’s effect on CLL B-cells showed that high-dose of AA induces cytotoxicity in CLL B-cells [21, 22]. Moreover, some CLL patients suffer from vitamin C deficiency (hypovitaminosis C), which is correlated with more aggressive disease [23]. Given that CLL B-cells are known to be sensitive to oxidative stress mediated by H_2_O_2_ [24–27], we hypothesized that a redox-inducing agent like AA might effectively kill leukemic cells and synergize with CLL treatments. Here, we performed a comprehensive study of the in vitro effect of 250 μM AA on primary highly purified CLL B-cells and two cell lines. We investigated the intrinsic and extrinsic mechanisms that lead to AA-cytotoxicity and resistance, and we evaluated the effect of combining AA with a panel of FDA-approved drugs. Overall, our study provides detailed mechanistic insights into AA’s action on CLL B-cells and identified drug combination strategies that might enhance the efficacy of treating CLL B-cells.

## Methods

### Patients

Chronic lymphocytic leukemia B-cells were isolated from the peripheral blood of 40 treatment-naïve patients diagnosed according to international guidelines (Binet stage A) (Table S1). Only treatment-naïve Binet stage A patients were included in our study, given that treatment might have altered their B-cells’ response to ascorbic acid. Normal B-cells were isolated from donor lymphocyte infusions provided by age-matched healthy volunteers. Patients and healthy volunteers provided their written informed consent to participate to the study. The study was performed in accordance with the principles expressed in the Declaration of Helsinki. This study was conducted in compliance with French legislation on non-interventional studies.

### Reagents

L-Ascorbic acid, dehydroascorbic acid (DHA), AA 2-phosphate (Asc-2P), catalase from human erythrocytes, deferoxamine (DFX), oligomycin A, and metformin were purchased from Sigma Aldrich. Sodium pyruvate (SP) was purchased from Thermo Fisher Scientific. Venetoclax (ABT-199), ibrutinib, idelalisib, fludarabine, and cyclophosphamide were purchased from Selleckchem. CPI-613 was purchased from Abcam.

### Cell isolation and cell culture

Peripheral blood mononuclear cells (PBMCs) were isolated by Ficoll density gradient centrifugation. CD19+ CD5+ B-cells were isolated from PBMCs using magnetic-bead-activated cell sorting, using a B-CLL cell isolation kit (Miltenyi Biotec). To evaluate the effect of AA on normal lymphocytes, naïve B-cells were isolated from donor lymphocyte infusions using a Naïve B-cell Isolation kit (Miltenyi Biotech). The purity assessed by CD19 expression on flow cytometry was around 98%. OSU-CLL cells were a gift from E. Hertlein and colleagues [28]. JVM3 cells were purchased from the Deutsche Sammlung von Mikroorganismen und Zellkulturen (DSMZ). Freshly isolated B-cells and CLL cell lines were cultured in RPMI 1640 medium (PAN Biotech, #P04–16500) with 10% fetal bovine serum (FBS) (PAN Biotech, #P30–3306) and L-glutamine, penicillin and streptomycin (1%) (Eurobio Scientific). When indicated, CLL B-cells were cultured in Iscove’s modified Dulbecco’s Medium (IMDM) (Merck, #FG0465) and alpha-MEM medium (Sigma Aldrich, #M4526) (*n* = 7). Cells were cultured at a density of 4 × 10^5^/ml in 48-well plates and were treated with either vehicle or AA or drugs for 24 h. To simulate hypoxia condition, cells were cultured in presence of 100 μM Cobalt(II) chloride hexahydrate (CoCl_2_.6H_2_O, Sigma Aldrich) for 24 h. Cells were then washed and incubated with AA for 24 h.

### Cell viability assay

Cell viability and apoptosis were assessed using annexin V-APC/ 7-AAD staining (BD Biosciences). Fluorescence intensity was measured in a MACSQuant Analyzer (Miltenyi Biotech). Data were analyzed using Flow Jo software (version 10, Tree Star, Inc.). Cell viability of treated cells was normalized to vehicle condition (i.e. cell viability of vehicle treated cells for each patient is set to 100% and all data were indicated relative to this value). In some experiment (when indicated, ex. in drugs combination experiments), cell viability was assessed using CellTiter-Glo Luminescent Cell Viability Assay Kit (Promega).

### Co-culture conditions with primary human bone marrow mesenchymal stem cells (MSCs)

MSCs were isolated from healthy donor bone marrow, as described by Naudot et al. [29]. MSCs were seeded at 2 × 10^4^ cells/ml in 24-well plates (Falcon) in alpha minimum essential medium (MEM) supplemented with 10% FBS, penicillin/streptomycin (1%), L-glutamine (1%) and 0.5 ng/ml basic fibroblast growth factor (bFGF) and incubated overnight to allow cells adhesion. Freshly isolated CLL B-cells were cultured alone or on MSCs at 4x10^5^cell/ml (ratio: 20:1) in RPMI medium. CLL cells were co-cultured with MSCs for 6 h (*n* = 12) or 24 h (*n* = 6) prior to AA treatment (250 μM). After 24 h, CLL cells were carefully removed and cell viability was assessed as described above.

CLL B-cells were stimulated with CpG-ODN2006 (1.5 μg/ml) (Invivogen), CD40L (50 ng/ml) + IL-4 (50 ng/ml) (Miltenyi) or anti-IgM antibody (10 μg/ml) (Jackson ImmunoResearch) (*n* = 7) or cultured in the presence of a combination of cytokines (as described in [30]) (*n* = 6) or in presence of 10% of the autologous patient’ serum (*n* = 10) and treated with AA for 24 h before cell viability/apoptosis was assessed in an annexin V-APC/7-AAD flow cytometry assay.

### Detection of ROS

OSU-CLL and JVM3 cell lines (2 × 10^5^ cells) were treated with either vehicle or AA (250 μM) for 6 h and incubated with MitoSox™ (Thermo Fisher Scientific), a mitochondrial superoxide indicator, at 5 μM for 10 min at 37 °C (*n* = 7) according to the manufacturer’s user guide. Cells were then washed and fluorescence was analyzed with MACSQuant Analyzer (Miltenyi Biotech). Cells treated with H_2_O_2_ (50 μM) and AA (1 mM) were used as positive control. The fold change of mean fluorescence intensity (MFI) was then calculated in treated cells relative to controls.

### Measurement of the extracellular H_2_O_2_ concentration

Extracellular H_2_O_2_ (i.e. H_2_O_2_ in the medium) was measured after treatment with different concentrations of AA and at different time points in the presence or absence of catalase and SP, using the Pierce™ Quantitative Peroxide Assay Kit (Aqueous) (Thermo Fisher Scientific) according to the manufacturer’s user guide (*n* = 6). Absorbance was read in a GloMax® Discover Microplate Reader (Promega).

### Glutathione levels

Levels of total cellular glutathione (GSH), oxidized form glutathione (GSSG) and the GSH/GSSG ratio were measured using the GSH/GSSG-Glo™ assay (Promega) in the OSU-CLL and JVM3 cell lines 2 h after treatment with 250 μM AA (*n* = 3). Luminescence was read using a GloMax® Discover Microplate Reader (Promega). Data were presented as the GSH/GSSG ratio.

### mRNA extraction and gene expression analysis

Highly purified CLL B-cells, healthy donor B-cells (HD B-cells) and OSU-CLL and JVM3 cell lines were washed in PBS. mRNA was extracted using RNeasy Mini Kit (Qiagen), and 1 μg was reverse-transcribed using a High Capacity cDNA Reverse Transcription Kit (Thermo Fisher Scientific). The relative mRNA expression of catalase was analyzed in a qPCR assay with TaqMan Universal PCR Master Mix (Thermo Fisher Scientific). The housekeeping genes beta-actin and GADPH were used as endogenous controls in the expression analyses. All PCR reactions were performed in triplicate. The TaqMan Gene Expression assays for catalase (Assay ID Hs00156308_m1), GAPDH (Hs02786624_g1), and β-actin (Hs01060665_g1) were purchased from Thermo Fisher Scientific.

### Catalase knockdown with siRNA

MISSION® esiRNA human CAT and Control SiRNA (esiRNA targeting Renilla luciferase (RLUC)) were purchased from SIGMA Aldrich. 1 × 10^6^ JVM3 cells were transfected with CAT siRNA or Ctrl SiRNA (188 nM) using Amaxa® Cell Line Nucleofector® Kit V (Lonza, Germany), program T-016. Transfected JVM3 cells were cultured in 12-well plate (1 × 10^6^ cells/well) under standard culture conditions. Changes in catalase expression in JVM3 transfected cells were analyzed at 48 and 72 h post-transfection by western blot. At 72 h, the cells were then treated with ascorbic acid at different doses (250, 500 and 1000 μM) and were analyzed for cell viability by the CellTiter-Glo Luminescent Cell Viability Assay Kit (Promega).

### Western blots

The cells were washed with PBS and lysed in RIPA buffer (Sigma). Cell lysates were centrifuged at 14000 rpm for 5 min, and supernatants were collected. After determination of the protein content in a BCA assay (Thermo Fisher Scientific), 50 μg of protein were separated using 10% SDS-PAGE and was transferred onto nitrocellulose membranes (Thermo Fisher Scientific). The membranes were incubated overnight at 4 °C with antibodies against cleaved and total poly-ADP-ribose polymerase (PARP, #9542; 1:1000, Cell Signaling Technology), catalase (#sc-271,803; 1:100, Santa Cruz Biotechnology), cleaved caspase-3 (#9664; 1:1000, Cell Signaling Technology), cleaved caspase-8 (#9496; 1/1000 Cell Signaling Technology), cleaved caspase-9 (#7237; 1/1000, Cell Signaling Technology), HIF-1α (#sc-13,515; 1:200, Santa Cruz Biotechnology) or β-actin (#sc-47,778; 1:500, Santa Cruz). Blots were then washed with TBS-buffer with 0.2% Tween and incubated with secondary antibodies against rabbit (Thermo Fisher Scientific), mouse (Sigma) or goat (Santa Cruz) antibodies (1:2500). Blots were developed using SuperSignal™ West Pico PLUS Chemiluminescent Substrate (Thermo Fisher Scientific).

### Drugs combination with AA study and synergism determination

Synergism was evaluated with the Chou-Talalay Combination Index (CI) using the experimental design as recommended by Chou TC [31]. Drugs were serially diluted in culture media and then cells were added to the media (in two replicates) and incubated for 24 h. Cells were seeded in 96-well plates at 1 × 10^5^ cells per well. A constant molar ratio combination for drugs based on lethal concentration 50 (LC50) values was used. The cytotoxicity of these drugs or combinations was assessed by CellTiter-Glo assay (Promega). The combination indexes (CIs) and fraction affected (Fa) based on the Chou-Talalay method using CompuSyn software (CompuSyn Inc. Paramus, NJ, USA). CI values < 1 were considered as synergistic [31]. For the data in Fig. 7b, synergism was determined using the coefficient of drug interaction (CDI) [32, 33], which was calculated as CDI = AB/(A × B), where AB is the ratio of the two-drug combination group to the control group, and A or B is the ratio of the single-drug group to the control group. CDI values < 0.7 were considered as synergistic.

### Statistical analysis

Data were expressed as the mean ± standard error of the mean (SEM). The data are expressed as a percentage with respect to that of vehicle-treated cells (control), which was set to 100%. However, statistical analyses were done with absolute viability data. All statistical analyses were performed with GraphPad Prism® software (version 5.0; GraphPad Software Inc., San Diego, CA, USA). Statistical significance was assessed in a one-way analysis of variance. A two-sided paired t-test was used to detect significant differences between groups. The threshold for statistical significance difference was set to *p* < 0.05 (* *p* values < 0.05; ** *p* values < 0.01 and ***p values < 0.001). No blinding and no randomization of samples were applied.

## Results

### Ascorbic acid induces apoptosis in primary CLL B-cells and OSU-CLL cell line as a result of extracellular and intracellular H_2_O_2_ generation

**Fig. 1:**
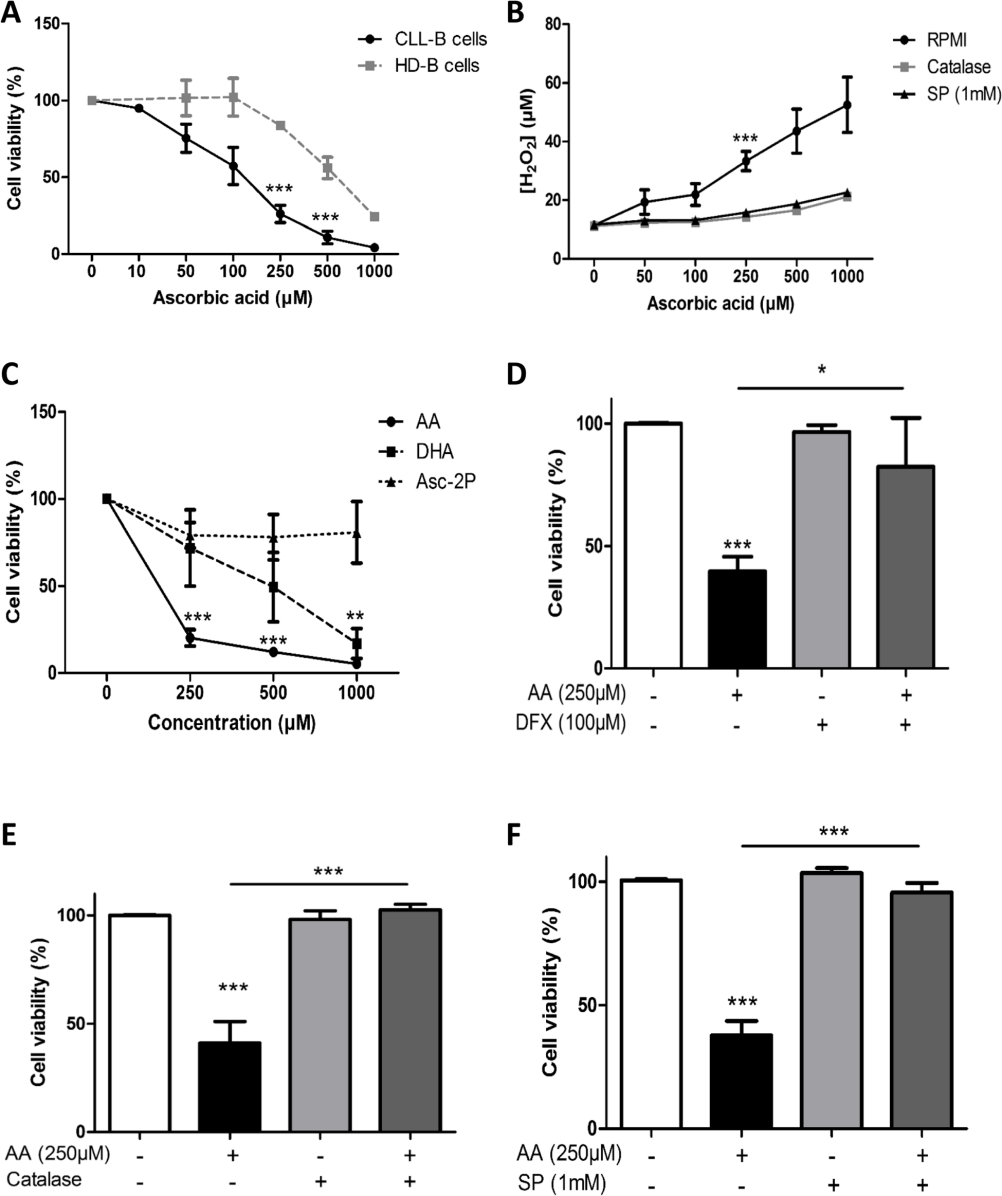
Ascorbic acid selectively kills CLL B-cells and has low toxicity toward B-cells from healthy donors (HD B-cells). Ascorbic acid’s effects are due to H_2_O_2_ generation and are reversed by catalase, sodium pyruvate (SP 1 mM) and the iron chelator deferoxamine (DFX). **a**: Viability of primary HD B-cells and CLL B-cells after 24 h of treatment with various concentrations of AA (***: *p* < 0.001 vs. Ctrl, CLL *n* = 16, HD B-cells *n* = 4). **b**: Extracellular H_2_O_2_ levels in the culture medium (RPMI) after 4 h of treatment with different concentrations of AA in the presence or absence of catalase (600 U/ml) or SP (1 mM) (*n* = 6). **c**: Primary CLL B-cell viability after 24 h of treatment with AA, dehydroascorbic acid (DHA) or AA 2-phosphate (Asc-2P); (*n* = 3) (**: *p* < 0.01, ***: *p* < 0.001 vs. Ctrl). **d**, **e** and **f**: Viability of CLL B-cells after 24 h of treatment with 250 μM AA in the presence or absence of DFX (100 μM) (*: *p* < 0.05; *n* = 5) (**d**), catalase (**: *p* < 0.01; *n* = 10) (**e**) and SP (1 mM) (***: *p* < 0.001; *n* = 20) (**f**). Data are presented as mean ± SEM

To assess the presence or absence of a cytotoxic effect of AA on CLL B-cells and HD B-cells, we treated freshly isolated cells with increasing concentrations of AA, determined the percentage of viable cells in an annexin V/7-AAD assay, and calculated the lethal concentration 50 (LC_50_) after 24 h. As shown in Fig. 1a, apoptosis was induced in a dose-dependent manner. For CLL B-cells, the LC_50_ was 213 μM - a dose that could be achieved in vivo by oral administration of the maximal tolerated amount of AA [16, 18]. In contrast, the LC_50_ for HD B-cells was 800 μM (Fig. 1a). When cells were incubated with 250 μM of AA, the cell survival rate was significantly lower than in control experiments (26 ± 16% for CLL B-cells (*p* < 0.001) and 84.4 ± 1% for HD B-cells) (Fig. 1a).

The major mechanism of AA cytotoxic activity in cancer cells is known to be auto-oxidation, which generates extracellular H_2_O_2_ (AscH + O_2_ → Asc^•^ + O_2_^•−^ → DHA + H_2_O_2_) [12]. Here, we found a dose-dependent elevation in the extracellular H_2_O_2_ concentration (i.e. in the CLL B-cells’ culture medium) after AA treatment (Fig. 1b). This elevation was inhibited by the H_2_O_2_ scavengers catalase [34] and 1 mM SP [35, 36] (Fig. 1b). We observed that 250 μM AA resulted in an H_2_O_2_ concentration of 40 μM. This concentration of H_2_O_2_ induced CLL cell death in vitro (Fig. S1A) but showed no significant effect on healthy donors B-cells (Fig. S1B).

In order to confirm that the observed cytotoxic effect of AA was caused by H_2_O_2_ generation, we treated CLL cells with a form of ascorbate that does not generate extracellular H_2_O_2_ (AA 2-phosphate (Asc-2P) and AA’s oxidation product (dehydroascorbic acid (DHA)). Neither Asc-2P nor DHA induced apoptosis in CLL B-cells after 24 h of treatment at a concentration of 250 μM. However, treatment with 1 mM DHA induced apoptosis in CLL B-cells (*p* < 0.01 vs. Ctrl) (Fig. 1c). Longer incubation times (48 h and 72 h) with DHA or Asc-2P at a concentration of 250 μM had no significant effect on primary CLL B-cells or OSU-CLL cell’s viability (Fig. S1C and D).

To further confirm the role of H_2_O_2_ in AA-mediated cytotoxicity, CLL B-cells were pre-incubated with the iron chelator deferoxamine (DFX) (which inhibits H_2_O_2_ generation [37]) at a concentration of 100 μM for 3 h and then treated with 250 μM AA or co-treated with AA and the H_2_O_2_ scavengers catalase or sodium pyruvate (SP). The pre-incubation of CLL B-cells with DFX inhibited the cytotoxic effect of AA (Fig. 1d). Incubation with catalase and SP also inhibited the cytotoxic effect of AA (Fig. 1e, f).

Sodium pyruvate is a constituent of some culture media and is also commonly added to other cell culture media as a carbon source in addition to glucose [38]. As we used pyruvate-free RPMI-1640 for our initials experiments, we decided to test the effect of AA on CLL B-cells cultured in two pyruvate-containing media: Iscove’s modified Dulbecco’s Medium (IMDM) and alpha-MEM. We observed that the cytotoxic effect of AA was absent when CLL B-cells were cultured in these media, relative to SP-free RPMI-1640 (Fig. S1E). Furthermore, the concentration of H_2_O_2_ generated by AA treatment was significantly lower in these media (Fig. S1F). These observations are in line with a previous study [39] on medium-dependent differences in AA’s ability to produce H_2_O_2_, and might explain some of the conflicting reports about the effect of AA on cancer cells in vitro [8].

### Ascorbic acid induces redox alteration in OSU-CLL cell line

**Fig. 2:**
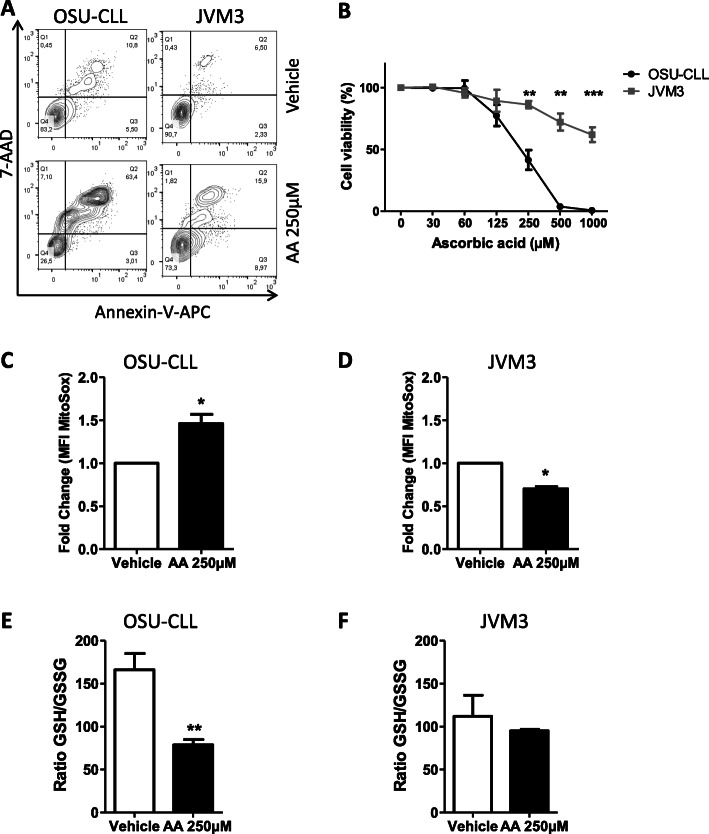
The JVM3 CLL cell line is less-sensitive to AA’s effects than the OSU-CLL cell line. **a**: Viability of OSU-CLL and JVM3 cells, assessed in a flow cytometry assay using annexin-V-APC and 7-AAD staining after treatment with vehicle or 250 μM AA. **b**: Viability of JVM3 and OSU-CLL cell lines after treatment with increased concentrations of AA (**: *p* < 0.01, ***: *p* < 0.001 vs. OSU-CLL at the same concentrations; *n* = 5). **c**, **d**: Mitochondrial ROS levels, as recorded in a MitoSox flow cytometry assay in OSU-CLL (**c**) and JVM3 cell lines (**d**) treated for 6 h with 250 μM AA and expressed as the fold change vs. vehicle (*: *p* < 0.05 vs. vehicle; *n* = 7). Data are presented as the fold change in mean ± SEM fluorescence intensity (MFI) for the MitoSox dye. **e**, **f**: The GSH/GSSG ratio in OSU-CLL (**e**) and JVM3 (**f**) cell lines was assessed after 2 h of treatment with 250 μM AA or vehicle, *: *p* < 0.05, **: *p* < 0.01 vs. vehicle (*n* = 3)

Given that H_2_O_2_ is membrane-permeant [40], we next measured levels of intracellular H_2_O_2_ accumulation in two human cell lines (OSU-CLL [28] and JVM3 [41]). As in the experiments on primary CLL cells, AA induced apoptosis in OSU-CLL cells, with an LC_50_ of 243.4 μM. In contrast, the JVM3 cell line was resistant to AA effects (Fig. 2a, b).

Mitochondrial superoxide radical levels were measured in a MitoSox flow cytometry assay. In OSU-CLL cells, the mean MitoSox fluorescence intensity was 38% higher for 250 μM AA than for the control (*p* < 0.05) (Fig. 2c and Fig. S2A). This difference was not observed in the presence of 1 mM SP (data not shown). In treated JVM3 cells, the MitoSox level decreases by 30% after AA treatment (*p* < 0.05) (Fig. 2d and Fig. S2B). Overall, these findings emphasize the central role of the oxidative stress induced by extracellular and intracellular H_2_O_2_ in the AA-mediated cytotoxicity against CLL B-cells.

In order to confirm redox alteration induced by AA treatments in cell lines, we assessed total and oxidized levels of GSH (a key intracellular antioxidant) in OSU-CLL and JVM3 cell lines after 2 h treatment with 250 μM AA. We observed significantly lower GSH/GSSG ratios than in control experiments (*p* < 0.01), indicating GSH oxidation in the OSU-CLL cell line after treatment with AA (Fig. 2e) but no difference was observed in JVM3 cell line (Fig. 2f). We next measured the H_2_O_2_ concentration in the extracellular medium from JVM3 cell cultures 4 h after AA treatment. We observed that 250 μM AA significantly increased the H_2_O_2_ concentration in the culture medium (*p* < 0.05) (Fig. S2C), which suggests that differences in the cell lines’ intrinsic characteristics affect the sensitivity to AA.

### Catalase expression affects the CLL B-cells’ response to AA

**Fig. 3:**
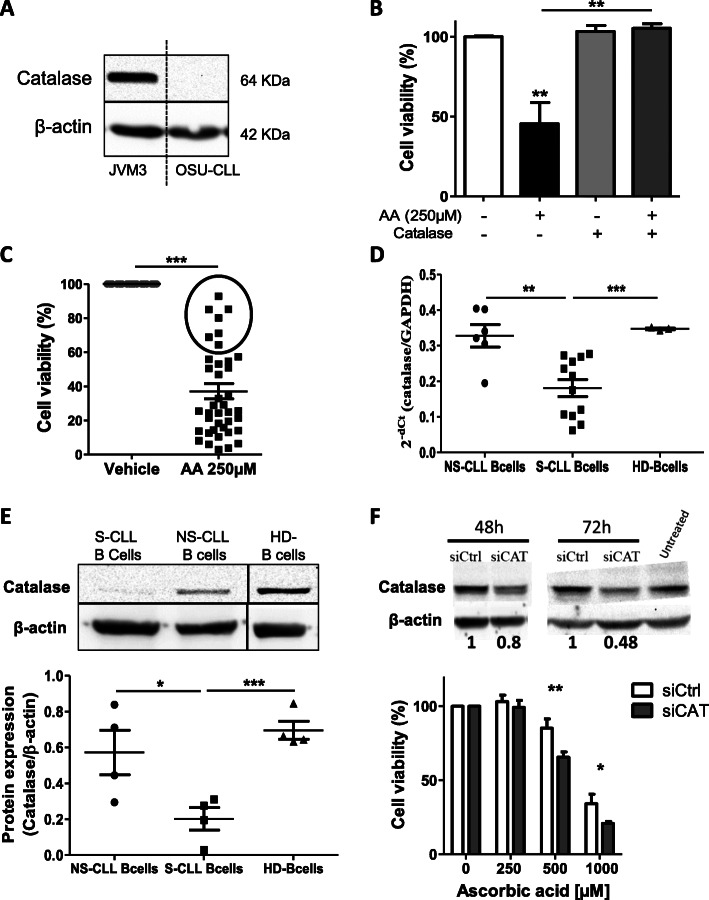
CLL B-cells’ sensitivity to AA is altered by catalase expression. **a**: catalase protein expression in OSU-CLL and JVM3 cell lines. **b**: Viability of OSU-CLL cells after AA treatment for 24 h in the presence or absence of catalase (600 U/ml) (**: *p* < 0.01; *n* = 6). **c**: Viability of primary CLL B-cells after treatment with AA for 24 h (***: *p* < 0.001; *n* = 40). **d**: Relative mRNA expression of catalase vs. GAPDH in CLL B-cells from AA-sensitive patients (S-CLL B-cells) or AA-non-sensitive patients (NS-CLL B-cells) and B-cells from healthy donors (HD B-cells). **e**: Catalase protein expression (normalized against β-actin) in HD B-cells and CLL B-cells (*: *p* < 0.05, ***: *p* < 0.001). **f**: Upper panel: Western blot and quantification of catalase protein levels following treatment of JVM3 cells for 48 h and 72 h with a control siRNA (siCtrl) or siRNA against catalase (siCAT). Relative catalase protein levels were quantified using ImageJ software. Lower panel: At 72 h, siRNA-transfected cells were treated by AA and cell viability was assessed after 24 h using the CellTiter-Glo Luminescent Cell Viability Assay Kit (*n* = 2 in duplicate). *: *p* < 0.05, **: *p* < 0.01. Data are presented as the mean ± SEM

As the JVM3 cell line was less sensitive than the OSU-CLL cell line to AA, we analyzed the cells’ expression of antioxidant enzymes (catalase and peroxiredoxin 1 (PRDX1)) (Fig. S2D). Unlike OSU-CLL cells, JVM3 cells express high levels of catalase (Fig. 3a and Fig. S2D). We also found that the AA-induced cytotoxicity in primary CLL B-cells and in the OSU-CLL cell line was inhibited by exogenous catalase (Fig. 1e and Fig. 3b). Next, we hypothesized that elevated catalase expression might account for the observed resistance to AA in primary CLL B-cells. Indeed, among the 40 patient samples tested, a group of 7 (17.5%) were found to be less sensitive to AA (i.e. > 50% cell viability after AA treatment) than the 33 others (Fig. 3c). We then compared catalase expression in sensitive vs. non-sensitive (resistant) B-cells by qPCR and Western blot assays. We observed significantly lower catalase mRNA expression (*p* < 0.01) (Fig. 3d) and significantly lower catalase protein level in sensitive CLL B-cells than in non-sensitive cells (*p* < 0.05) (Fig. 3e and Fig. S2E). Furthermore, catalase expression was significantly lower in CLL B-cells than in HD B-cells (*p* < 0.001) (Fig. 3d, e).

To assess whether the catalase is directly implicated in the resistance to AA, JVM3 cells were transfected with catalase specific-siRNAs to knock-down catalase expression. Cells were analyzed at 48 h and 72 h post-transfection for catalase expression by Western blotting. Compared to the control, the expression of catalase was reduced by the half at 72 h (Fig. 3f). Therefore, AA was added to the culture at 72 h post-transfection and cell viability was assessed 24 h later. In the absence of AA treatment, the downregulation of catalase in JVM3 cells had no effect on cell viability. However, in presence of AA at 500 μM and 1000 μM, a significant reduction of cell viability was observed in catalase knock-down cells compared to control (Fig. 3f), suggesting that catalase play a role in the resistance of cells to AA treatment.

Hence, we concluded that catalase expression by CLL B-cells might be involved in their resistance to AA.

### Ascorbic acid kills CLL cells through caspase-dependent apoptosis

**Fig. 4:**
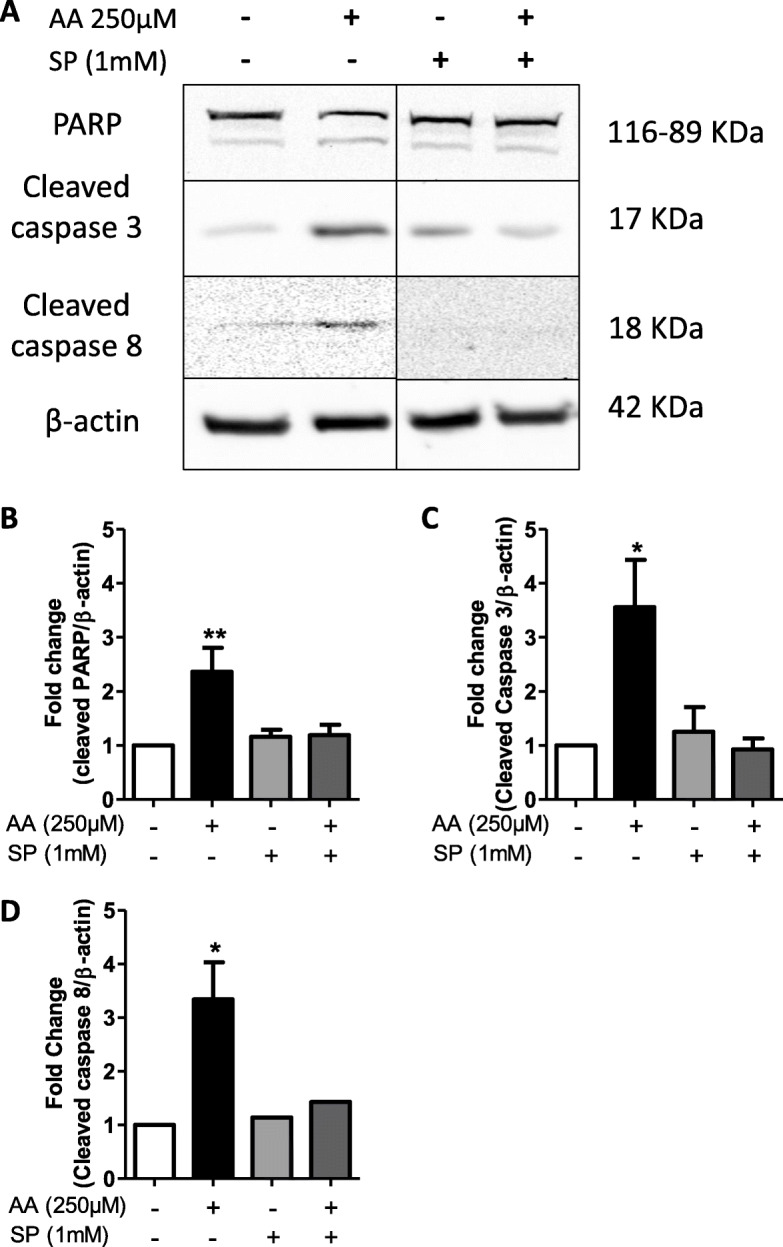
AA-induced apoptosis is caspase-dependent. **a**: Western blot of PARP cleavage, cleaved caspase-3, cleaved caspase-8, and β-actin in OSU-CLL cells treated with 250 μM AA for 6 h in the presence or absence of sodium pyruvate (SP, 1 mM). **b**, **c**, and **d**: Quantification of cleaved PARP (*n* = 5) (B), cleaved caspase-3 (*n* = 5) (**c**) and cleaved caspase-8 (*n* = 3) (**d**), normalized against β-actin. Data are presented as the fold-change vs. the control (mean ± SEM) (*: *p* < 0.05, **: *p* < 0.01 vs. Ctrl)

To study the molecular mechanism of AA-induced cell death, we analyzed the activation of the caspase cascade (particularly caspases-3, − 8, and − 9) and the active caspase target PARP in the OSU-CLL cell line. We found that AA treatment induced PARP cleavage in OSU-CLL cells (1.9-fold vs. ctrl) (*p* < 0.01) (Fig. 4a, b). Furthermore, 250 μM AA induces caspase-3 in OSU-CLL cells (3.5-fold vs. the control, *p* < 0.05) (Fig. 4a, c). Levels of cleaved caspase-8 were significantly higher in AA-treated OSU-CLL cells (*p* < 0.05) (Fig. 4a, d). These effects were inhibited by SP; incubation with SP restored caspase activity to baseline levels - indicating that the generated H_2_O_2_ induced cells killing via a caspase pathway.

### AA overcome the supporting effect of the microenvironment on CLL B-cells

Signals from the CLL microenvironment support the survival of CLL B-cells and has a critical role in the cells’ drug resistance [42]. We decided to study how these signals impact AA’s cytotoxic effects.

**Fig. 5:**
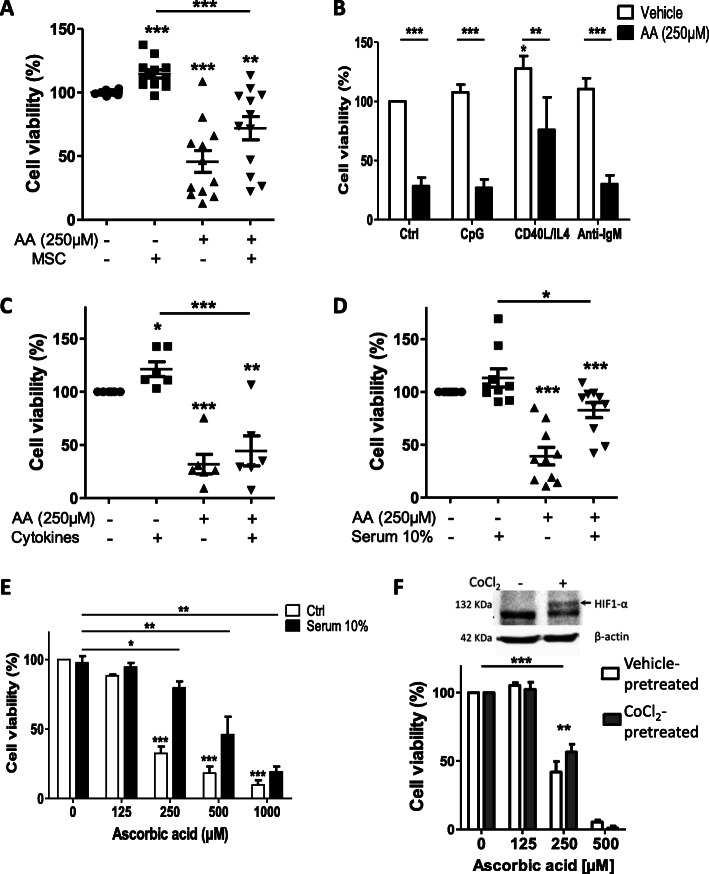
Effects of the microenvironment on AA-induced apoptosis in CLL B-cells. **a**: CLL cells were co-cultured for 6 h with MSCs prior to AA treatment (250 μM). After 24 h of AA treatment, CLL cells were collected and analyzed for cell viability by an annexin V/7AAD staining (**: *p* < 0.01, ***: *p* < 0.001; *n* = 12). **b**: Effects of AA on cell viability in the presence of CD40L + IL-4, CpG or anti-IgM (*: *p* < 0.05, **: *p* < 0.01, ***: *p* < 0.001 vs. Ctrl; *n* = 7). **c**: CLL B-cell viability in the presence or in absence of a combination of cytokines after 24 h of treatment with 250 μM AA (*: *p* < 0.05, **: *p* < 0.01, ***: *p* < 0.001 vs. vehicle; *n* = 6). **d**: Effects of 250 μM AA on the viability of CLL B-cells cultured in the presence of autologous patient serum (10%) or 10% FBS (Ctrl) (*: *p* < 0.05; ***: *p* < 0.001 vs. Ctrl; *n* = 10). **e**: Effects of increased concentrations of AA on the viability of CLL B-cells cultured in the presence of 10% autologous serum (*: *p* < 0.05; **: *p* < 0.01; ***: *p* < 0.001 vs. Ctrl; *n* = 5). **f**: OSU-CLL cells were pre-treated for 24 h with CoCl_2_ (100 μM) then incubated with different concentrations of AA. Upper panel: Western blot analysis showing HIF-1α levels under normoxia and CoCl_2_-induced hypoxia conditions. Lower Panel: Effect of AA on cell viability was assessed by the CellTiter-Glo cell viability assay (**: *p* < 0.01; ***: *p* < 0.001; *n* = 3 in duplicate). Data are presented as the mean ± SEM

We analyzed AA’s effects on CLL B-cells co-cultured with primary human bone marrow MSCs. This cell type is known to provide survival support to CLL B-cells [43, 44] and to protect CLL B-cells from oxidative stress [45, 46]. Before adding AA, CLL B-cells were co-cultured with MSCs for 6 h (Fig. 5a) or 24 h (Fig. S3) and cell viability was analyzed 24 h after the addition of AA. Our results evidenced greater viability of CLL cells in co-culture with MSCs (relative to CLL cells cultured alone; *p* < 0.001) (Fig. 5a and Fig. S3). However, AA treatment was still associated with significant killing of CLL B-cells in the presence of MSCs (Fig. 5a; *p* < 0.01 for MSC + AA vs. ctrl and *p* < 0.001 vs. MSCs) and Fig. S3.

We also analyzed the effects of AA in the presence of microenvironment stimulation by CD40L and interleukin (IL)-4 (that mimic T-cell help), CpG-ODN, or anti-IgM (that mimic antigenic stimulation). We observed greater viability of CLL cells stimulated with CD40L and IL-4, relative to the control (*p* < 0.05). Ascorbic acid treatment resulted in significantly lower viability of CD40L/IL-4 stimulated CLL B-cells (*p* < 0.01). Anti-IgM and CpG-ODN stimulation did not alter AA effects on CLL B-cells (Fig. 5b).

Next, CLL B-cells were treated with AA in the presence of a mixture of cytokines (IL-2, − 6, − 10, − 15, and − 21, BAFF, and APRIL) [30], and cell viability was assessed after 24 h. As expected [30], the presence of cytokines enhanced the viability of CLL B cells (*p* < 0.05) (Fig. 5c). We observed that in the presence of cytokines, cell viability of AA treated CLL B-cells was significantly lower than for nontreated cells (*p* < 0.001) (Fig. 5c).

We have reported previously that autologous serum from a CLL patient improved the survival of CLL B-cells in vitro [30]. Furthermore, H_2_O_2_ scavengers such as catalase and pyruvate are present in human serum [36, 40, 47]. We therefore studied the serum’s dose-effect relationship for AA. Primary B-cells were cultured with 10% autologous serum (rather than FBS, the control) and treated with 250 μM AA. Although autologous serum lowered AA’s cytotoxic effect, AA still induced significant cell death in the presence of 10% autologous serum (*p* < 0.05) (Fig. 5d). We then compared the effects of increasing concentrations of AA on CLL B-cell viability in the presence of 10% autologous serum. At a concentration of 500 μM, AA induced a high level of CLL B-cell death – even in the presence of patient serum (*p* < 0.01 vs. Ctrl) (Fig. 5e). These data strongly suggest that components in the CLL patient’s serum attenuate the AA’s effects on leukemic cells.

As CLL B-cells are in perpetual transition between normoxic compartment (the peripheral blood) and hypoxic tissues in lymph nodes and bone marrow, we next decided to study the impact of hypoxia on the cytotoxic effect of AA [46, 48]. To address this question, we used cobalt chloride (CoCl_2_) as a hypoxia-mimetic agent. OSU-CLL and JVM3 cell lines were pretreated with CoCl_2_ for 24 h and then AA was added and cell viability was measured 24 h later. We first checked by western blot the expression of the Hypoxia-inducible factor-1 alpha (HIF-1α) whose expression is activated in hypoxic condition. We observed that HIF-1α expression was effectively induced by CoCl_2_ treatment in OSU-CLL (Fig. 5f) and JVM3 cell lines (Fig. S3B). At 250 μM AA, the OSU-CLL cell line showed a greater viability in CoCl_2_-pretreated cells relative to control (*p* < 0.01) (Fig. 5f); however, AA treatment was still associated with significant killing of these cells (*p* < 0.001) (Fig. 5a). Nevertheless, CoCl_2_-pretreated JVM3 cells showed no significant changes in their response to AA (Fig. S3B).

Overall, these data suggest that AA-mediated cytotoxicity overcome the protective effect of the microenvironment.

### In vitro, AA synergizes with several drugs used to treat CLL

**Fig. 6:**
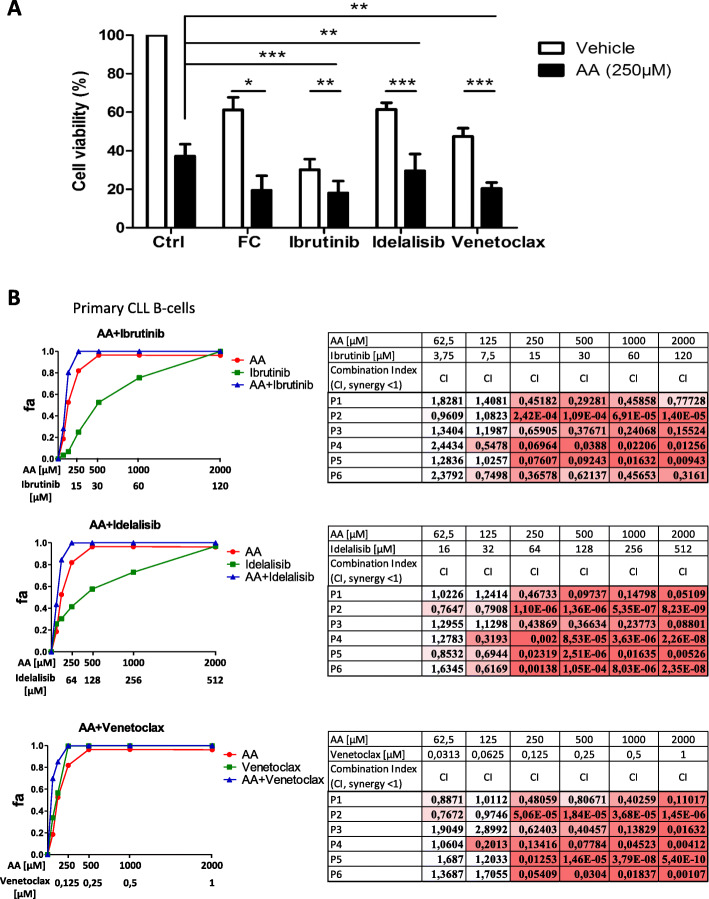
Ascorbic acid synergistically increases the effects of targeted therapies on CLL B-cells. **a**: Viability of CLL B-cells treated with 250 μM AA alone or in combination with the approved drugs fludarabine (35 μM) and cyclophosphamide (100 μM) (*n* = 6), ibrutinib (15 μM) (*n* = 11), idelalisib (50 μM) (*n* = 12), and venetoclax (10 nM) (*n* = 16)) (*: *p* < 0.05; **: *p* < 0.01, ***: *p* < 0.001). Cell viability was determined by an annexin V/7AAD staining. **b**: Synergistic efficacy of combination of AA and CLL targeted therapies in primary CLL B-cells. Left panels: Primary CLL cells (*n* = 6) were treated with ascorbic acid (AA) and ibrutinib or idelalisib or venetoclax for 24 h. CellTiter-Glo cell viability assay was performed to detect cell kill synergy. The curves show the dose-effect of single drugs and that of drugs combination. Each data point is the mean of six samples. Right panels: Tables show the combination index (CI) of each combination for each patient (P). The CI values were calculated using the Chou-Talalay method by the software Compusyn. Fa: fraction affected (fraction of cells affected by a particular drug dose)

We next investigated the interaction between AA and (i) FDA-approved drugs for the treatment of CLL and other hematological malignancies (fludarabine + cyclophosphamide, ibrutinib, idelalisib, and venetoclax) or the treatment of solid cancers (CPI-613), and (ii) molecules that are in preclinical development (oligomycin A and metformin). We found that AA enhanced the cytotoxic effect of ibrutinib, idelalisib, and venetoclax to kill primary CLL B-cells (*p* < 0.001, *p* < 0.05 and *p* < 0.01, respectively) (Fig. 6a). Similarly, AA potentiates venetoclax’s cytotoxic effect on OSU-CLL cells (venetoclax alone vs. venetoclax+AA; *p* < 0.001) (Fig. S4A). OSU-CLL cells treated with the venetoclax-AA combination showed slightly higher levels of PARP and caspase-3 cleavages than cells treated with venetoclax alone; however, this difference was not statistically significant (Fig. S4B, C, D). The combinations of these drugs with AA at the same doses had no significant impact on healthy donors’ B-cells survival (Fig. S5).

To study synergism between AA and CLL targeted therapies (ibrutinib, idelalisib and venetoclax), we used the Chou-Talalay method in a constant-ratio combination design [31]. To identify synergistic effects, the combination index (CI) was calculated by the software Compusyn (CI < 1). The single-agent dose-effect curves of primary CLL B-cells from 6 patients are shown in Fig. S6. The combination index values calculated using the actual experimental data points showed synergistic interactions (CI < 1) between AA and all three drugs at AA dose of 250 μM or higher for all 6 patients (Fig. 6b). For several patients, synergism was observed at 250 μM of AA but also at lower doses (Fig. 6b). Similar experiments were conducted using OSU-CLL and JVM3 cell lines (Fig. S7A and B). Interestingly, in both cell lines, AA showed synergy in combination with venetoclax at 250 μM AA (Fig. S7A and B).

**Fig. 7:**
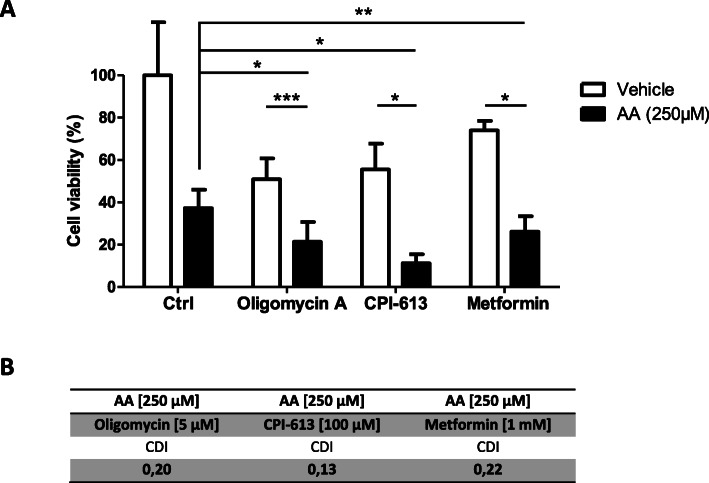
Ascorbic acid synergistically potentiates the effects of mitochondrial metabolism targeting therapies on CLL B-cells. **a**: Effects of metabolic enzyme inhibitors oligomycin A (5 μM), CPI-613 (100 μM) and metformin (1 mM) on the viability of CLL B-cells after treatment alone or in combination with 250 μM AA for 24 h (*: *p* < 0.05, **: *p* < 0.01, ***: *p* < 0.001; *n* = 5). Cell viability was determined by an annexinV/7AAD staining. Data are presented as the mean ± SEM. **b**: Table show the coefficient of drug interaction (CDI) for drug combination with AA showed in (**a**). CDI values < 0.7 were considered as synergistic

It is known that CLL B-cells depend primarily on oxidative phosphorylation for energy generation, and that oxidative phosphorylation is associated with greater ROS production [49]. We hypothesized that targeting mitochondrial metabolism might enhance AA’s cytotoxicity in CLL cells. We examined the interaction between AA and a number of currently implemented therapeutic approaches that target mitochondrial metabolism, with regard to CLL B-cells viability. Treatment with CPI-613 (an alpha ketoglutarate dehydrogenase inhibitor), metformin (a respiratory chain complex I inhibitor) or oligomycin A (an ATP synthase inhibitor) was associated with significantly lower CLL B-cell viability (*p* < 0.05, *p* < 0.01 and *p* < 0.01, respectively) (Fig. 7a) and calculation of the coefficient of drug interaction (CDI) indicated a synergistic effect (CDI < 0.7) (Fig. 7b). To study synergism between AA and these drugs in OSU-CLL and JVM3 cell lines, we applied the Chou-Talalay experimental model [31] (Fig. S8). Synergistic interactions (CI < 1) between AA and all three drugs were observed in both cell lines (Fig. S8). However, in OSU-CLL cell line, AA and Oligomycin A combination showed synergism at 250 μM of AA but also at lower doses and in JVM3 cell line, AA and Metformin combination showed synergism at 250 μM of AA but also at lower doses (Fig. S8). Overall, these data suggest that combining AA and mitochondria-targeting drugs including venetoclax, metformin and oligomycin A is promising therapeutic approach in CLL.

## Discussion

Despite major progress in treatment, CLL is still an incurable disease. We wondered whether oral supplementation with AA (vitamin C) would have an impact on CLL B-cell survival. To study this effect in vitro, we used recent knowledge from pharmacokinetic studies showing that oral administration of AA can achieve a plasma concentration of around 250 μM [16, 18]. Using this concentration, we (i) provided mechanistic insights into the cytotoxic effect of AA on CLL B-cells, (ii) investigated the role of the CLL microenvironment in resistance to AA’s effect, and (iii) suggested new therapeutic strategies. We showed that 250 μM AA selectively induced cell death in primary leukemic CLL B-cells by acting as a pro-oxidant and thus by leading to the release of H_2_O_2_ into the extracellular medium. These results are in compliance with previous observations in other cancers [5, 12, 14]. The cytotoxic effect of AA via H_2_O_2_ generation was confirmed using an H_2_O_2_ formation inhibitor (the iron chelator DFX) and two H_2_O_2_ scavengers (catalase and SP). These molecules completely reversed AA’s cytotoxicity toward CLL B-cells. Knowing that H_2_O_2_ can enter the cell through passive diffusion [4], we observed high intracellular and mitochondrial ROS levels and low GSH/GSSG ratios in AA-treated CLL cells vs. the control - confirming the presence of a redox alteration in the treated cells. On the molecular level, we showed that AA treatment induces cleavage of caspase-8 but not caspase-9; this suggests that apoptosis was extrinsically mediated and not mitochondrial [50, 51]. Along with the superoxide anion (O_2_^•−^) and the hydroxyl radical (^•^OH), H_2_O_2_ is a ROS. The literature data show that ROS (including H_2_O_2_) mediate apoptosis through increasing the level of oxidative stress in cancer cells [14, 15, 52]. Indeed, targeting intracellular redox homeostasis by increasing ROS levels in cancer cells is a promising treatment approach [15]. CLL B-cells were shown to produce abnormally large amounts of ROS and to have impaired antioxidant defenses [24, 27, 53]. Hence, these cells are vulnerable to molecules that perturb redox homeostasis [46, 49].

Catalase is an anti-oxidant enzyme that protects normal cells against AA-mediated oxidative stress by degrading the generated H_2_O_2_ [26, 34, 54]. Consequently, normal cells remove AA-generated H_2_O_2_ faster than tumor cells do [55]. As shown here and by others [56], CLL B-cells express lower levels of catalase than normal B-cells do. Nevertheless, we found that CLL B-cells from 7 of 40 patients (17.5%) were less sensitive to AA treatment; all these cases showed high levels of catalase expression. Importantly, it has been shown that CLL B-cells expressing high levels of catalase lead to a more aggressive disease [56]. The role of catalase was further emphasized by the observed AA-resistance of the catalase-expressing JVM3 cell line; in contrast, the OSU-CLL cell line (which does not express catalase) was sensitive to AA. The knockdown of catalase expression in JMV3 cells sensitized these cells to the cytotoxic effect of AA indicating that catalase plays a role in the resistance to AA.

Recent studies have shown that the CLL microenvironment (including bone marrow MSCs) not only provides survival cues [30, 57] but also protects against oxidative stress by modulating the expression of genes involved in redox homeostasis and by increasing glutathione synthesis [24, 45, 46]. However, our present results show that AA was able to induce CLL B-cell death and thus overcome the anti-apoptotic protection provided by primary bone marrow MSCs. Furthermore, AA was also able to counter the survival support provided by another microenvironment cue from T cells (modeled here by incubation with CD40L and IL4). Moreover, hypoxia is shown to provoke resistance toward anticancer drugs. In CLL, malignant cells recirculate from normoxic peripheral blood to hypoxic tissues like lymph nodes and bone marrow, where hypoxia was shown to provide survival advantage [46, 48]. We observed that chemically induced hypoxia by CoCl_2_ provided survival advantages to CLL cells in presence of 250 μM AA. These could be explained by the intracellular pyruvate accumulation caused by the fact that under hypoxic environment CLL cells shift from oxidative phosphorylation to glycolysis as a source of energy [46, 48]. Nevertheless, we were surprised to find that autologous serum protected against (but did not completely abolish) the cytotoxic effect of 250 μM AA. Furthermore, a still higher concentration of AA (500 μM) induced significant cell death in the presence of autologous serum. We have previously reported that culture of CLL B-cells with 10% autologous serum protected against apoptosis [30]. However, in the context of AA treatment, this protective effect can be explained by the presence of H_2_O_2_ scavengers (such as catalase and pyruvate) in the human serum [27, 36, 40, 47]. Indeed, plasma catalase activity was higher in CLL patients than in healthy subjects [27] and was correlated with the disease’s aggressiveness. Here, we also showed that SP protects CLL B-cells from the cytotoxic effect of AA. Furthermore, SP has been demonstrated to directly protect CLL cells against oxidative stress and to increase cell viability after H_2_O_2_ treatment [48]. We confirmed this effect by showing that the concentration of AA-generated H_2_O_2_ in the medium falls in the presence of SP. The pyruvate concentration in human plasma is around 100 μM, and this concentration increases after glucose uptake because pyruvate is a major product of glycolysis [35, 58, 59]. Furthermore, glucose was shown to inhibit intestinal vitamin C transport ex vivo [60], and blood glucose levels may interfere with the uptake of ascorbate by human neutrophils [61]. These observations suggest that considering adequate dietary patterns is critical when delivering AA supplements orally. Hence, AA-induced cytotoxicity might be enhanced by a glucose-restricted diet that could increase intestinal AA uptake and reduce plasma pyruvate concentrations. On the same lines, recent data showed a synergistic effect between fasting-mimicking diet and vitamin C in KRAS mutated colorectal cancer [6]. Moreover, our data showed that the culture medium influenced the CLL B-cells’ response to AA treatment. The cytotoxic effect of AA observed in basic RPMI 1640 medium was completely absent when cells were cultured in IMDM and alpha-MEM – both of which contain pyruvate as built-in component. This observation underlines the critical role of the cell culture medium in the cancer cells’ response to AA and may account for the conflicting results in the literature [7, 8]. Importantly, our results suggest that publications about AA’s effect in vitro should always specify the exact reference of the cell culture medium and/or the exact compound added to the medium.

Given that previous studies generated conflicting findings on the influence of vitamin C on cancer treatments [8], we investigated the compound’s influence on currently available drugs for CLL and hematological malignancies and drugs in the development pipeline. We observed that AA synergistically potentiates the cytotoxicity of ibrutinib, idelalisib, and venetoclax in primary CLL B-cells. Potentiation was also observed for drug candidates like CPI-613 (an alpha-ketoglutarate dehydrogenase inhibitor), the ATP synthase inhibitor oligomycin A, and metformin.

In CLL cells, perturbations in oxidative metabolism result in elevated levels of ROS; this is associated with a favorable prognosis and slower disease progression [62]. The generation of oxidative stress might be useful for treating cancer directly or for enhancing sensitivity to other cancer drugs. It has been reported that BH3 mimetics can displace Bcl-2-bound glutathione, which thus inhibits the transport of glutathione into mitochondria and makes the cell more vulnerable to oxidative stress [63–65]. In line with these previous observations, we observed synergistic CLL cell killing by a combination of venetoclax and AA. Mechanistically, the data suggest that this synergistic effect is linked to downregulation of *MCL1* expression by the two treatments. MCL-1 is an anti-apoptotic protein involved in resistance to venetoclax and ibrutinib. Trachootham et al. [66] have shown that ROS decrease the expression of MCL-1 in CLL cells by inhibiting its glutathionylation. Therefore, the decrease in MCL-1 expression associated with AA–induced ROS favors the use of a combination therapy with venetoclax and AA. This finding might be of value in designing rational new treatment regimens by combining venetoclax with inducers of oxidative stress. Similarly, PI3K inhibition has been linked to increased oxidative stress in CLL cells through the inactivation of NRF2 [67]. This effect might combine with ROS to target MCL-1 because the protein is more stable after phosphorylation by AKT [68]. This might explain the synergistic cytotoxicity of idelalisib and AA for CLL B-cells. Furthermore, in addition to synergistic cytotoxic effect of ibrutinib/idelalisib with AA on CLL cells; ibrutinib and idelalisib induces the mobilization of leukemic cells from their protective tissue microenvironment to the blood circulation [2], leading to the loss of this protective effect, and CLL cells eventually becoming more susceptible to cell death by AA.

Given that metabolic activity in CLL cancer cells results in an altered redox state [69, 70], we decided to study the combination of ROS-inducing agent (i.e. AA) with drugs that targeting metabolic pathways such as the inhibitor of the tricarboxylic acid cycle CPI-613, the ATP synthase inhibitor oligomycin A and the electron transport chain complex I inhibitor metformin that target mitochondrial metabolism. CPI-613 and metformin are currently in clinical testing for hematologic malignancies including CLL [69, 71]. Furthermore, CPI-613, oligomycin A and metformin showed synergistic effects with AA in killing CLL B-cells; hence, the combination of AA with drugs targeting mitochondrial metabolism might be a promising approach in CLL treatment.

## Conclusion

In conclusion, our results show that AA at 250 μM induces apoptotic cell death of CLL B-cells in a caspase-dependent manner. This process involves the generation of reactive oxygen species in the extracellular media and in CLL cells. We also show that AA treatment overcome the supportive effect of the CLL microenvironment. Targeted therapies (idelalisib and venetoclax) effects could be enhanced by AA. Moreover, AA synergistically potentiates the cytotoxicity of several drugs that target mitochondrial metabolism. Indeed, the dose of AA used here for inducing apoptotic cell death in CLL B-cells could be achievable by oral administration of vitamin C. Therefore, vitamin C supplementation may be used as a novel combination therapeutic approach for the treatment of CLL.

## Supplementary information

**Additional file 1: Figure S1.** A: Viability of CLL B-cells 24 h after treatment with AA (250 μM) or H_2_O_2_ (40 μM), normalized against vehicle (**: *p* < 0.01; ***: *p* < 0.001 vs. vehicle; *n* = 3). B: Viability of healthy donor B-cells 24 h after treatment with H_2_O_2_ (40 μM), normalized against vehicle (*n* = 4). C: and D: Primary CLL B-cells (C, *n* = 5) and OSU-CLL cells (D, *n* = 3) viability after 24, 48 and 72 h of treatment with AA, dehydroascorbic acid (DHA) or AA 2-phosphate (Asc-2P) (*: *p* < 0.05; **: *p* < 0.01; ***: p < 0.001 vs. Ctrl). E: Viability of CLL B-cells in RPMI, IMDM or alpha-MEM culture media and in the presence of sodium pyruvate (SP 1 mM) (**: p < 0.01; ***: p < 0.001 vs. Ctrl; *n* = 7). F: The H_2_O_2_ concentration in RPMI or IMDM media 4 h after treatment with 250 μM AA (**: p < 0.01; *n* = 3). **Figure S2.** A, B: Mitochondrial ROS levels, as recorded in a MitoSox flow cytometry assay in OSU-CLL (A) and JVM3 cell lines (B) treated for 6 h with 250 μM AA, 1 mM AA or H_2_O_2_ (50 μM) and expressed as the fold change vs. vehicle (*: p < 0.05, **: p < 0.01 vs. ctrl). C: The H_2_O_2_ concentration in the culture medium of JVM3 cells 4 h after treatment with vehicle or 250 μM AA (*: p < 0.05 vs. vehicle, *n* = 3). D: mRNA relative expression of catalase and PRDX1 in OSU-CLL and JVM3 cell lines (*: p < 0.05 vs. OSU-CLL, *n* = 4). E: Western blot showing catalase protein expression in AA-sensitive patients (S-CLL B-cells) or AA-non-sensitive patients (NS-CLL B-cells) and B-cells from healthy donors (HD B-cells). **Figure S3.** A: CLL cells were co-cultured for 24 h with MSCs prior to AA treatment (250 μM). After 24 h of AA treatment, CLL cells were removed and analyzed for cell viability by an annexin V/7AAD staining (*: p < 0.05, *n* = 6). B: JVM3 cells were pre-treated for 24 h with CoCl_2_ (100 μM) then incubated with different concentrations of AA. Upper panel: Western blot analysis showing HIF-1α levels under normoxia and CoCl_2_-induced hypoxia conditions. Lower Panel: Effect of AA on cell viability was assessed by the CellTiter-Glo cell viability assay (***: p < 0.001; *n* = 3 in duplicate). Data are presented as the mean ± SEM. **Figure S4.** Ascorbic acid enhanced venetoclax’s effects on the OSU-CLL cell line. A: Viability of the OSU-CLL cell line treated with 250 μM AA alone or in combination with 10 nM venetoclax for 24 h. B: Cleavage of PARP and caspase-3 in OSU-CLL cells treated with AA alone or in combination with venetoclax for 6 h. C: Quantification of cleaved PARP, or cleaved caspase-3 (D), normalized against β-actin (*: p < 0.05; **: p < 0.01; *n* = 5). Data are presented as the mean ± SEM. **Figure S5.** Viability of healthy donor B-cells treated with 250 μM AA alone or in combination with the CLL’s approved drugs, ibrutinib (15 μM) idelalisib (50 μM) and venetoclax (10 nM)). *n* = 4. Data are presented as mean ± SEM. **Figure S6.** The dose-effect curves of single drugs in primary CLL cells from 6 patients (P) using the CellTiter-Glo cell viability assay. **Figure S7.** Synergistic efficacy of AA and CLL targeted therapies combination in OSU-CLL and JVM3 cell lines. A and B: left Panels: CellTiter-Glo assay was performed to detect cell kill synergy after 24 h in OSU-CLL and JVM3 cells treated with ascorbic acid (AA) and ibrutinib or idelalisib or venetoclax. The curves show the dose-effect of single drugs and of drugs combination. Each value is the mean of one experiment in duplicate. Right panels: Tables show the combination index (CI). The CI values were calculated using the Chou-Talalay method by the software Compusyn. Fa: fraction affected (fraction of cells affected by a particular drug dose). **Figure S8.** Synergistic efficacy of AA and CPI-613, oligomycin A or metformin in OSU-CLL and JVM3 cell lines. A and B: left Panels: CellTiter-Glo assay was performed to detect cell kill synergy after 24 h in OSU-CLL and JVM3 cells treated with ascorbic acid (AA) and CPI-613, oligomycin A or metformin. The curves show the dose-effect of single drugs and of drugs combination. Each value is the mean of one experiment in duplicate. Right panels: Tables show the combination index (CI). The CI values were calculated using the Chou-Talalay method by the software Compusyn. Fa: fraction affected (fraction of cells affected by a particular drug dose). **Table S1.** Features of the CLL patients included in the study. The CLL was staged according to Binet’s classification. Matutes score was based on the expression of CD5, CD79, CD23, FMC7, and sIg. IGHV mutational status homology ≥98%: UM, unmutated; V3–21*, a subset conferring a worse prognosis. Cytogenetic abnormalities were determined by karyotyping and/or FISH. The CLL patient’s karyotype was classified as follows: normal (no abnormalities detected), del (deletion), or ND (not determined). Response to ascorbic acid (AA) was determined by assessing cell viability after 24 h treatment with AA (250 μM) (i.e. resistant: > 50% cell viability after AA treatment).

## Data Availability

All data generated or analyzed during this study are included in this published article and its supplementary information files.

## Abbreviations

AA: Ascorbic acid
APRIL: A proliferation-inducing ligand
Asc-2P: Ascorbic acid 2-phosphate
BAFF: B-Cell-Activating Factor
BCL: B-cell lymphoma
BTK: Bruton’s tyrosine kinase
CAT: Catalase
CI: Combination index
CDI: Coefficient of drug interaction
CLL: Chronic lymphocytic leukemia
CoCl_2_: Cobalt chloride
CpG-ODN: CpG oligodeoxynucleotides
Ctrl: Control
Fa: Fraction affected
DFX: Deferoxamine
DHA: Dehydroascorbic acid
GSH: Reduced Glutathion
GSSG: Oxidized Glutathion
H_2_O_2_: Hydrogen peroxide
HD: Healthy donor
HIF: Hypoxia-inducible factor
IL: Interleukin
LC50: Lethal concentration 50
MCL1: Myeloid cell leukemia 1
μM: Micromolar
MSC: Mesenchymal stem cell
nM: Nanomolar
PBMCs: Peripheral blood mononuclear cells
PI3K: Phosphoinositol-3-kinase
PLCG2: Phospholipase C Gamma 2
PRDX1: Peroxiredoxin 1
ROS: Reactive oxygen species
siRNA: Small interfering RNA
SP: Sodium pyruvate
