# Supplementary material for: Ascorbic acid (vitamin C) synergistically enhances the therapeutic effect of targeted therapy in chronic lymphocytic leukemia
Source: J Exp Clin Cancer Res. 2020 Oct 28;39:228. doi: 10.1186/s13046-020-01738-0 (PMC7594454; doi:10.1186/s13046-020-01738-0)
Supplement: Supplementary file 1 — Additional file 1: Figure S1. A: Viability of CLL B-cells 24 h after treatment with AA (250 μM) or H2O2 (40 μM), normalized against vehicle (**: p < 0.01; ***: p < 0.001 vs. vehicle; n = 3). B: Viability of healthy donor B-cells 24 h after treatment with H2O2 (40 μM), normalized against vehicle (n = 4). C: and D: Primary CLL B-cells (C, n = 5) and OSU-CLL cells (D, n = 3) viability after 24, 48 and 72 h of treatment with AA, dehydroascorbic acid (DHA) or AA 2-phosphate (Asc-2P) (*: p < 0.05; **: p < 0.01; ***: p < 0.001 vs. Ctrl). E: Viability of CLL B-cells in RPMI, IMDM or alpha-MEM culture media and in the presence of sodium pyruvate (SP 1 mM) (**: p < 0.01; ***: p < 0.001 vs. Ctrl; n = 7). F: The H2O2 concentration in RPMI or IMDM media 4 h after treatment with 250 μM AA (**: p < 0.01; n = 3). Figure S2. A, B: Mitochondrial ROS levels, as recorded in a MitoSox flow cytometry assay in OSU-CLL (A) and JVM3 cell lines (B) treated for 6 h with 250 μM AA, 1 mM AA or H2O2 (50 μM) and expressed as the fold change vs. vehicle (*: p < 0.05, **: p < 0.01 vs. ctrl). C: The H2O2 concentration in the culture medium of JVM3 cells 4 h after treatment with vehicle or 250 μM AA (*: p < 0.05 vs. vehicle, n = 3). D: mRNA relative expression of catalase and PRDX1 in OSU-CLL and JVM3 cell lines (*: p < 0.05 vs. OSU-CLL, n = 4). E: Western blot showing catalase protein expression in AA-sensitive patients (S-CLL B-cells) or AA-non-sensitive patients (NS-CLL B-cells) and B-cells from healthy donors (HD B-cells). Figure S3. A: CLL cells were co-cultured for 24 h with MSCs prior to AA treatment (250 μM). After 24 h of AA treatment, CLL cells were removed and analyzed for cell viability by an annexin V/7AAD staining (*: p < 0.05, n = 6). B: JVM3 cells were pre-treated for 24 h with CoCl2 (100 μM) then incubated with different concentrations of AA. Upper panel: Western blot analysis showing HIF-1α levels under normoxia and CoCl2-induced hypoxia conditions. Lower Panel: Effect of AA on cell [file 13046_2020_1738_MOESM1_ESM.pdf]

Supplementary Figure 1

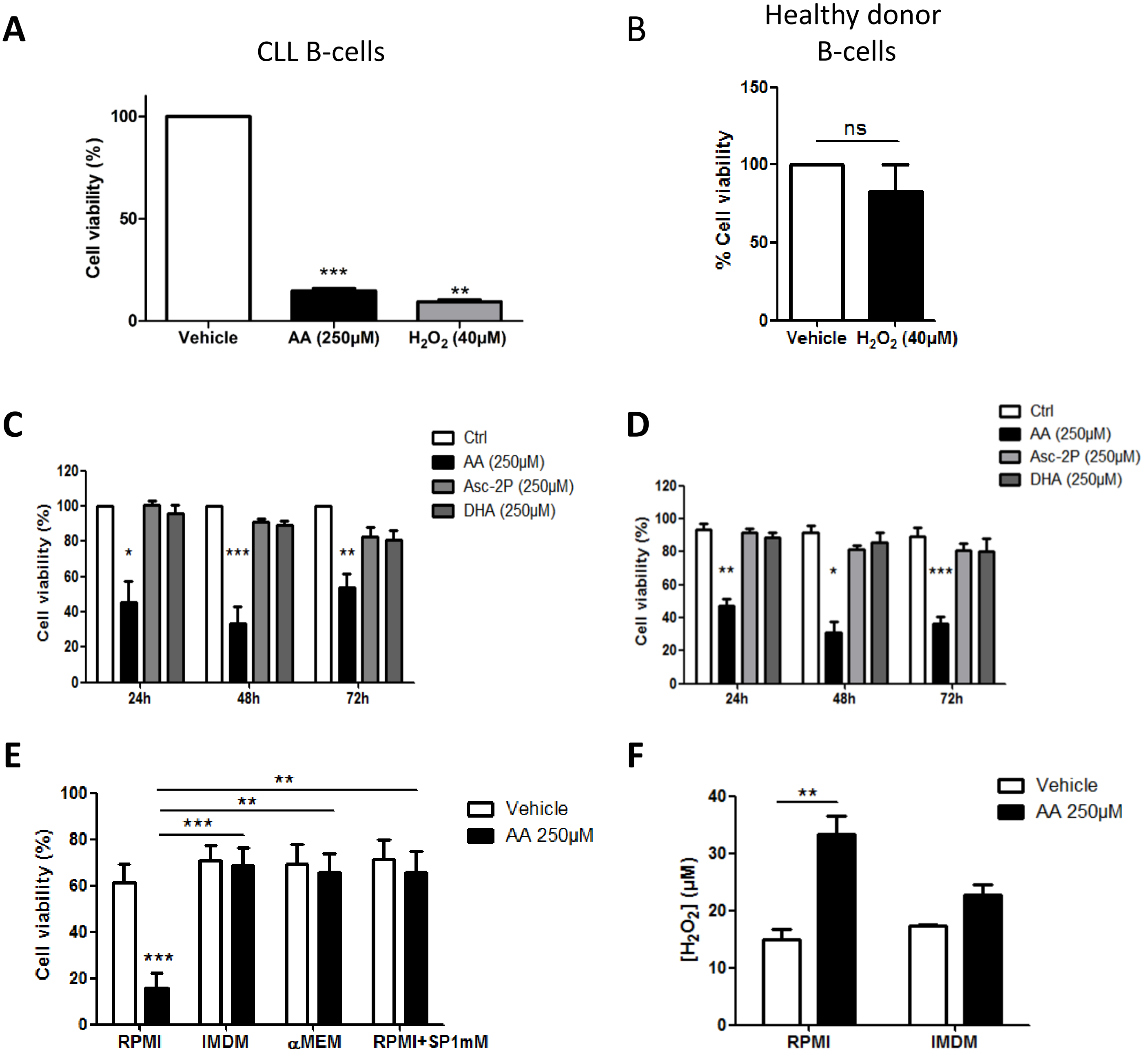


**Figure S1.** A: Viability of CLL B-cells 24 h after treatment with AA (250 μM) or $H_2O_2$ (40 μM), normalized against vehicle (**: $p<0.01$; ***: $p<0.001$ vs. vehicle; n=3). B: Viability of healthy donor B-cells 24 h after treatment with $H_2O_2$ (40 μM), normalized against vehicle (n=4). C: and D: Primary CLL B-cells (C, n=5) and OSU-CLL cells (D, n=3) viability after 24, 48 and 72 hours of treatment with AA, dehydroascorbic acid (DHA) or AA 2-phosphate (Asc-2P) (*: $p<0.05$; **: $p<0.01$; ***: $p<0.001$ vs. Ctrl). E: Viability of CLL B-cells in RPMI, IMDM or alpha-MEM culture media and in the presence of sodium pyruvate (SP 1 mM) (**: $p<0.01$; ***: $p<0.001$ vs. Ctrl; n=7). F: The $H_2O_2$ concentration in RPMI or IMDM media 4 h after treatment with 250 μM AA (**: $p<0.01$; n=3).

# Supplementary Figure 2

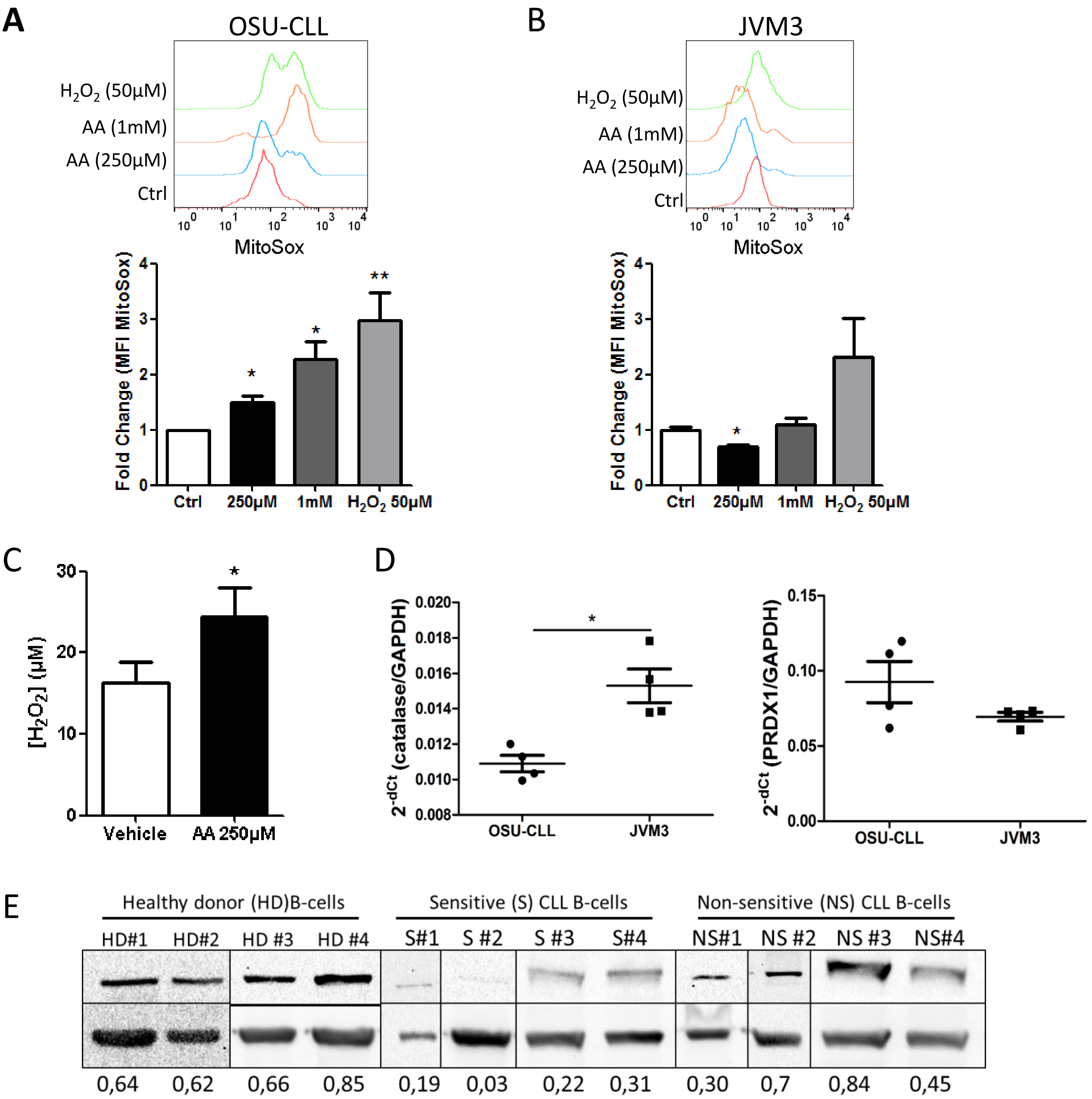


**Figure S2**. A, B: Mitochondrial ROS levels, as recorded in a MitoSox flow cytometry assay in OSU-CLL (A) and JVM3 cell lines (B) treated for 6 h with 250 µM AA, 1mM AA or $H_2O_2$ (50µM) and expressed as the fold change vs. vehicle (*: $p<0.05$, **: $p<0.01$ vs. ctrl). C: The $H_2O_2$ concentration in the culture medium of JVM3 cells 4 h after treatment with vehicle or 250 µM AA (*: $p<0.05$ vs. vehicle, n=3). D: mRNA relative expression of catalase and PRDX1 in OSU-CLL and JVM3 cell lines (*: $p<0.05$ vs. OSU-CLL, n=4). E: Western blot showing catalase protein expression in AA-sensitive patients (S-CLL B-cells) or AA-non-sensitive patients (NS-CLL B-cells) and B-cells from healthy donors (HD B-cells).

# Supplementary Figure 3

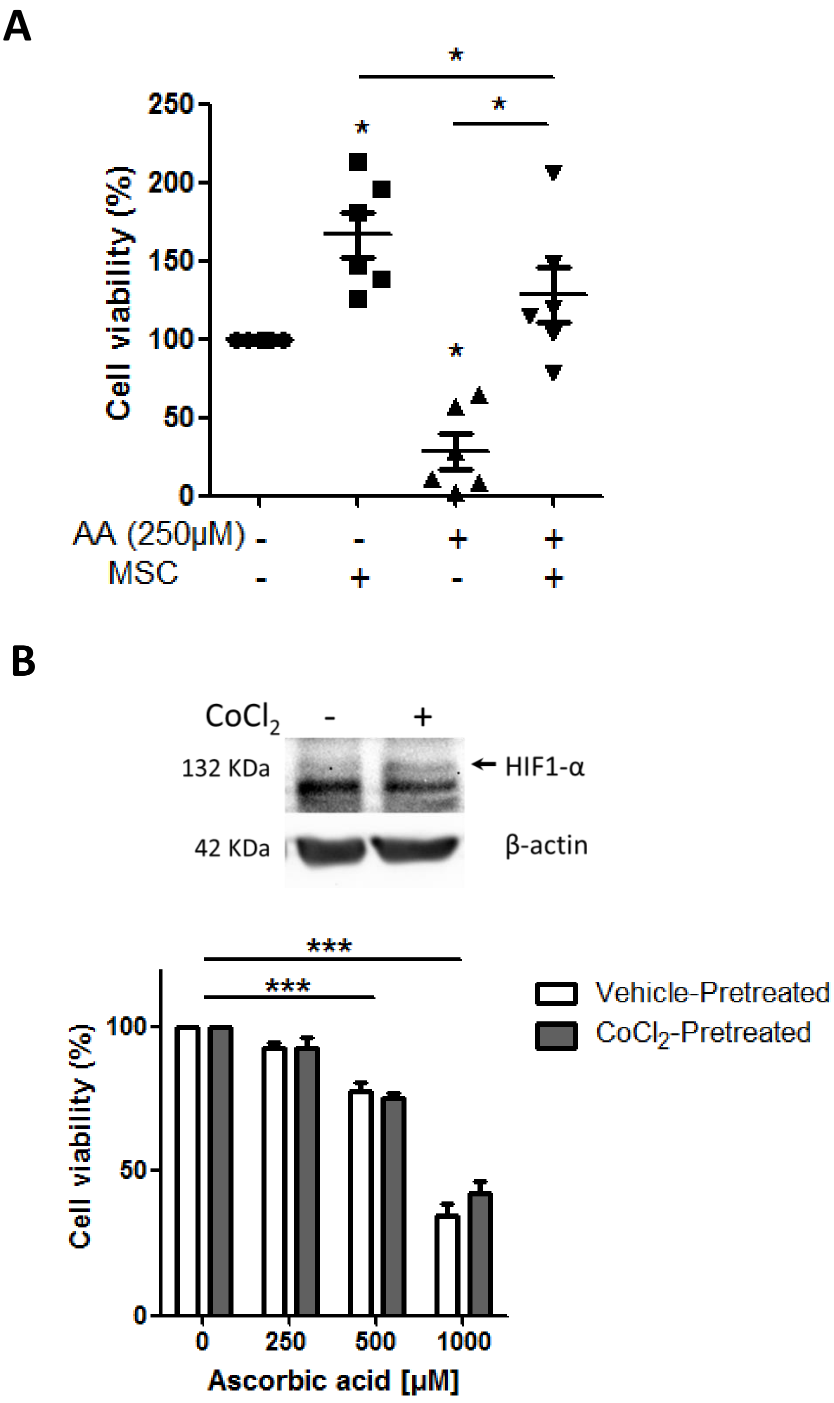


**Figure S3**. A: CLL cells were co-cultured for 24h with MSCs prior to AA treatment (250µM). After 24 h of AA treatment, CLL cells were removed and analyzed for cell viability by an annexin V/7AAD staining (*: p<0.05, n=6). B: JVM3 cells were pre-treated for 24h with $CoCl_2$ (100 µM) then incubated with different concentrations of AA. Upper panel: Western blot analysis showing HIF-1α levels under normoxia and $CoCl_2$-induced hypoxia conditions. Lower Panel: Effect of AA on cell viability was assessed by the CellTiter-Glo cell viability assay ( ***: p<0.001; n=3 in duplicate). Data are presented as the mean ± SEM.

# Supplementary Figure 4

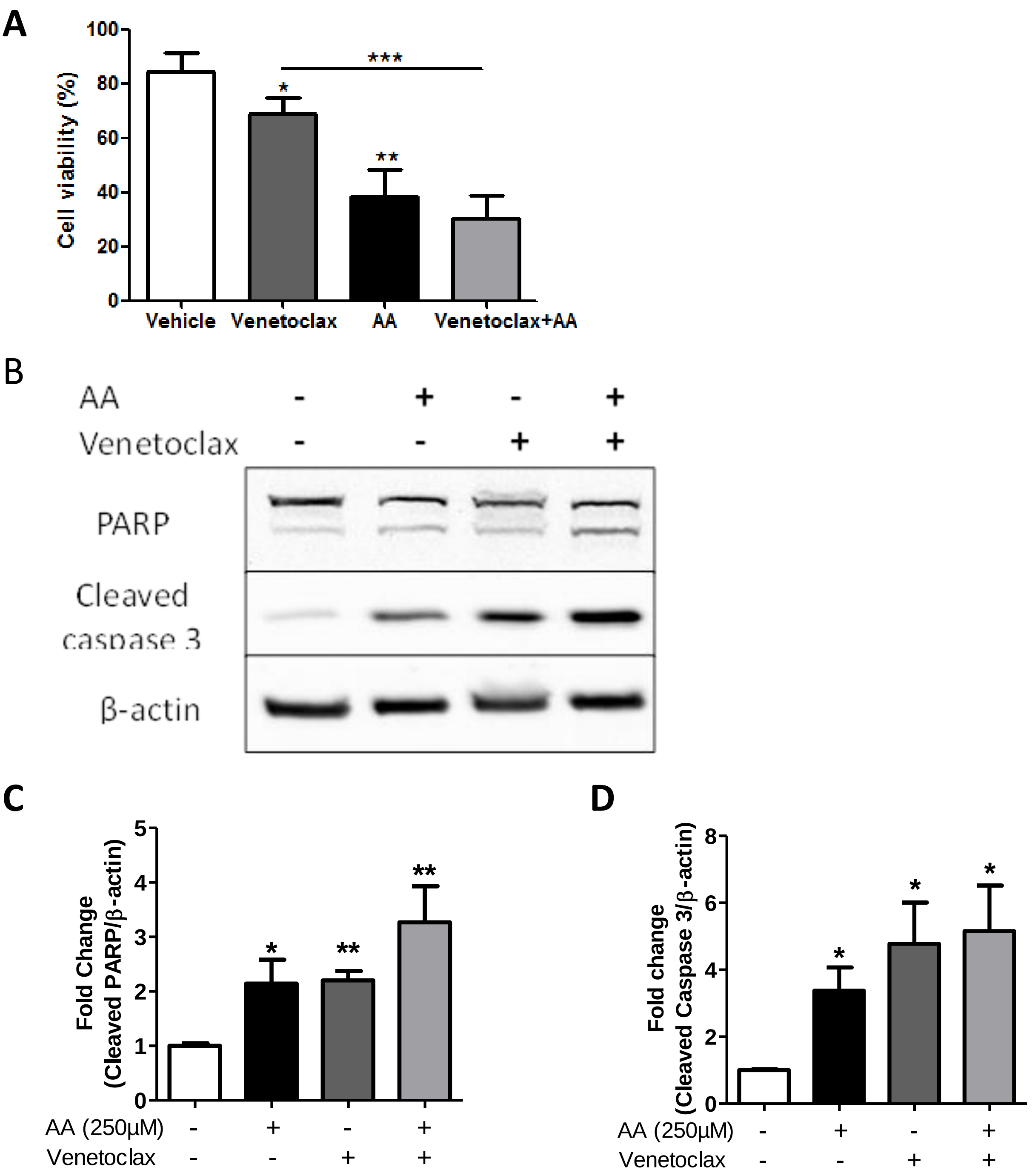


**Figure S4**. Ascorbic acid enhanced venetoclax's effects on the OSU-CLL cell line. A: Viability of the OSU-CLL cell line treated with 250 µM AA alone or in combination with 10 nM venetoclax for 24 h. B: Cleavage of PARP and caspase-3 in OSU-CLL cells treated with AA alone or in combination with venetoclax for 6 h. C: Quantification of cleaved PARP, or cleaved caspase-3 (D), normalized against β-actin (*: $p<0.05$; **: $p<0.01$; n=5). Data are presented as the mean ± SEM.

Supplementary Figure 5

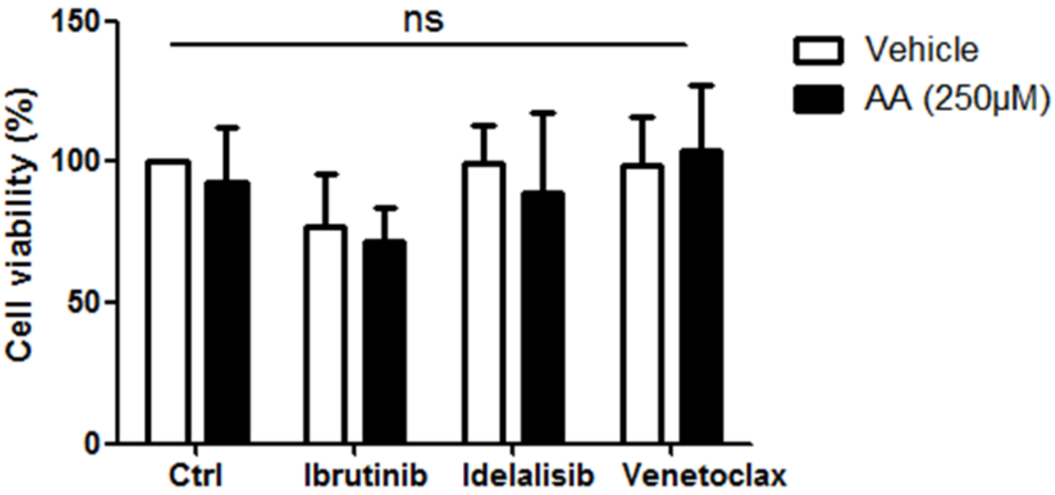


**Figure S5**. Viability of healthy donor B-cells treated with 250 µM AA alone or in combination with the CLL's approved drugs, ibrutinib (15 µM) idelalisib (50 µM) and venetoclax (10 nM)). n=4. Data are presented as mean ± SEM.

# Supplementary Figure 6

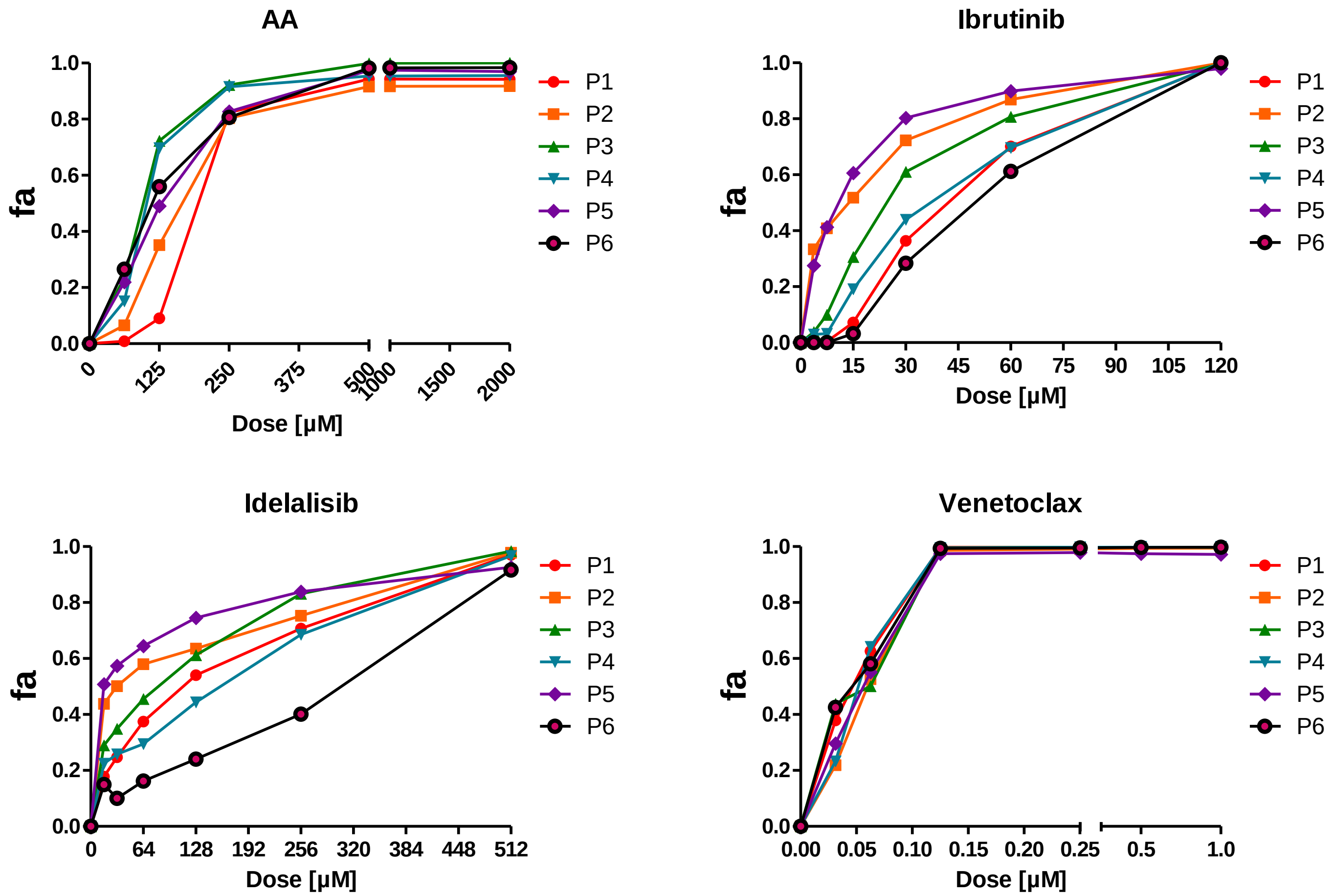


**Figure S6**. The dose-effect curves of single drugs in primary CLL cells from 6 patients (P) using the CellTiter-Glo cell viability assay.

# Supplementary Figure 7

## A

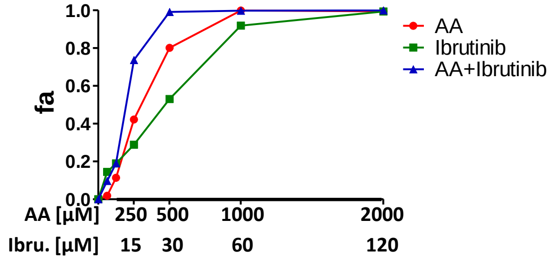


### OSU-CLL

| AA [µM] | 62,5 | 125 | 250 | 500 | 1000 | 2000 |
|---|---|---|---|---|---|---|
| Ibrutinib [µM] | 3,75 | 7,5 | 15 | 30 | 60 | 120 |
| Combination Index (CI, synergy <1) | CI | CI | CI | CI | CI | CI |
| OSU-CLL | **1,29782** | **1,83973** | **1,29069** | **0,60083** | **0,64987** | **0,33921** |

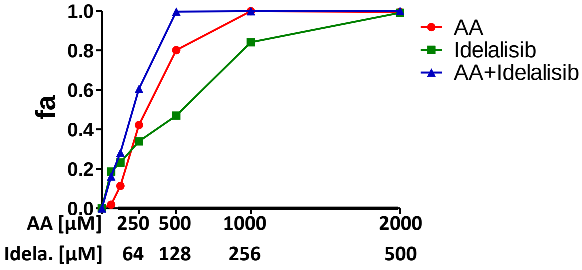


| AA [µM] | 62,5 | 125 | 250 | 500 | 1000 | 2000 |
|---|---|---|---|---|---|---|
| Idelalisib [µM] | 16 | 32 | 64 | 128 | 256 | 512 |
| Combination Index (CI, synergy <1) | CI | CI | CI | CI | CI | CI |
| OSU-CLL | **1,08984** | **1,53754** | **1,62092** | **0,40104** | **0,53979** | **0,29584** |

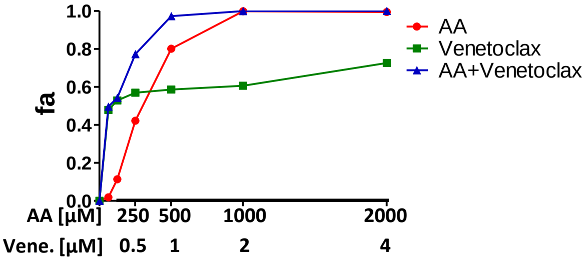


| AA [µM] | 62,5 | 125 | 250 | 500 | 1000 | 2000 |
|---|---|---|---|---|---|---|
| Venetoclax [µM] | 0,125 | 0,25 | 0,5 | 1 | 2 | 4 |
| Combination Index (CI, synergy <1) | CI | CI | CI | CI | CI | CI |
| OSU-CLL | **1,2278** | **1,11696** | **0,68846** | **0,63106** | **0,26791** | **0,11585** |

## B

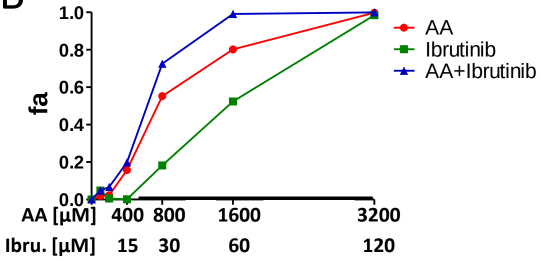


### JVM3

| AA [µM] | 100 | 200 | 400 | 800 | 1600 | 3200 |
|---|---|---|---|---|---|---|
| Ibrutinib [µM] | 3,75 | 7,5 | 15 | 30 | 60 | 120 |
| Combination Index (CI, synergy <1) | CI | CI | CI | CI | CI | CI |
| JVM3 | **0,67821** | **1,19247** | **1,53694** | **1,1979** | **0,68729** | 0,48061 |

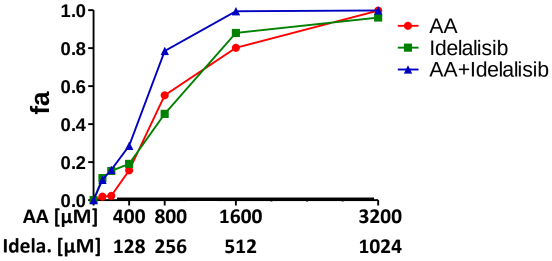


| AA [µM] | 100 | 200 | 400 | 800 | 1600 | 3200 |
|---|---|---|---|---|---|---|
| Idelalisib [µM] | 32 | 64 | 128 | 256 | 512 | 1024 |
| Combination Index (CI, synergy <1) | CI | CI | CI | CI | CI | CI |
| JVM3 | **1,00185** | **1,57095** | **2,12199** | **1,41523** | **0,50386** | **0,46581** |

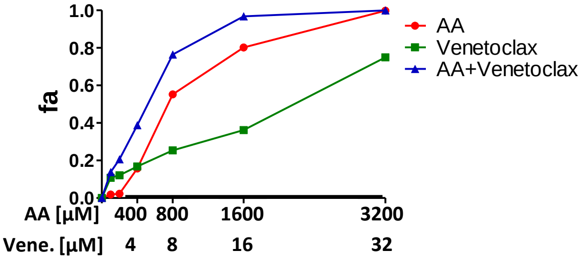


| AA [µM] | 100 | 200 | 400 | 800 | 1600 | 3200 |
|---|---|---|---|---|---|---|
| Venetoclax [µM] | 1 | 2 | 4 | 8 | 16 | 32 |
| Combination Index (CI, synergy <1) | CI | CI | CI | CI | CI | CI |
| JVM3 | **0,75117** | **0,93657** | **0,95439** | **0,93908** | **0,75922** | **0,32977** |

**Figure S7**. Synergistic efficacy of AA and CLL targeted therapies combination in OSU-CLL and JVM3 cell lines. A and B: left Panels: CellTiter-Glo assay was performed to detect cell kill synergy after 24 h in OSU-CLL and JVM3 cells treated with ascorbic acid (AA) and ibrutinib or idelalisib or venetoclax. The curves show the dose-effect of single drugs and of drugs combination. Each value is the mean of one experiment in duplicate. Right panels: Tables show the combination index (CI). The CI values were calculated using the Chou-Talalay method by the software Compusyn. Fa : fraction affected (fraction of cells affected by a particular drug dose).

# Supplementary Figure 8

A

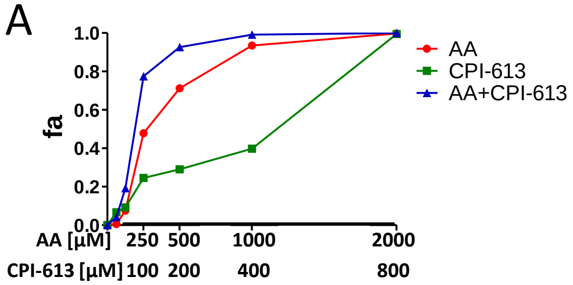


OSU-CLL

| AA [µM] | 62,5 | 125 | 250 | 500 | 1000 | 2000 |
|---|---|---|---|---|---|---|
| CPI-613 [µM] | 25 | 50 | 100 | 200 | 400 | 800 |
| Combination Index (CI, synergy <1) | CI | CI | CI | CI | CI | CI |
| OSU-CLL | **1,8062** | **1,43153** | **0,77282** | **0,86787** | **0,69406** | **0,18661** |

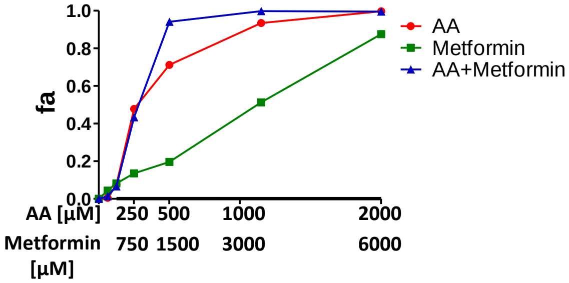


| AA [µM] | 62,5 | 125 | 250 | 500 | 1000 | 2000 |
|---|---|---|---|---|---|---|
| Metformin [µM] | 187.5 | 375 | 750 | 1500 | 3000 | 6000 |
| Combination Index (CI, synergy <1) | CI | CI | CI | CI | CI | CI |
| OSU-CLL | 2,59493 | 1,9938 | 1,18751 | 0,65534 | 0,45266 | 0,95836 |

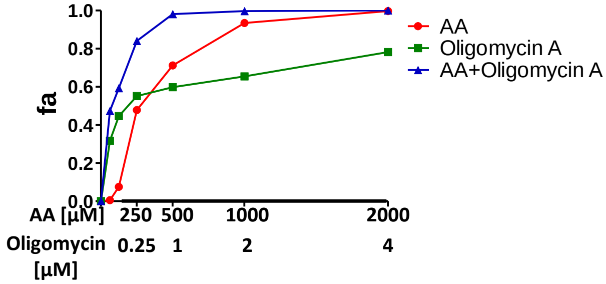


| AA [µM] | 62,5 | 125 | 250 | 500 | 1000 | 2000 |
|---|---|---|---|---|---|---|
| Oligomycin A [µM] | 0.0625 | 0.125 | 0.25 | 0.5 | 1 | 2 |
| Combination Index (CI, synergy <1) | CI | CI | CI | CI | CI | CI |
| OSU-CLL | 0,53936 | 0,60916 | 0,47045 | 0,38217 | 0,41298 | 4,32E-05 |

B

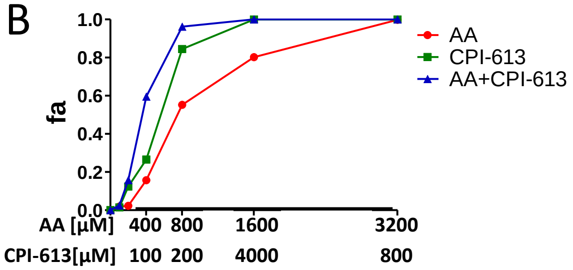


JVM3

| AA [µM] | 100 | 200 | 400 | 800 | 1600 | 3200 |
|---|---|---|---|---|---|---|
| CPI-613 [µM] | 25 | 50 | 100 | 200 | 400 | 800 |
| Combination Index (CI, synergy <1) | CI | CI | CI | CI | CI | CI |
| JVM3 | 1,29467 | 1,42938 | 1,57203 | 1,3993 | 0,0501 | 0,00175 |

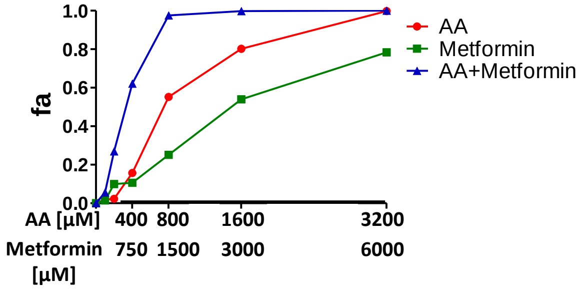


| AA [µM] | 100 | 200 | 400 | 800 | 1600 | 3200 |
|---|---|---|---|---|---|---|
| Metformin [µM] | 187,5 | 375 | 750 | 1500 | 3000 | 6000 |
| Combination Index (CI, synergy <1) | CI | CI | CI | CI | CI | CI |
| JVM3 | 0,91441 | 0,73045 | 0,73242 | 0,3855 | 0,28242 | 0,03513 |

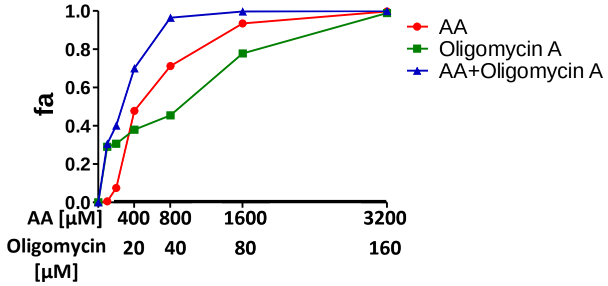


| AA [µM] | 100 | 200 | 400 | 800 | 1600 | 3200 |
|---|---|---|---|---|---|---|
| Oligomycin A [µM] | 5 | 10 | 20 | 40 | 80 | 160 |
| Combination Index (CI, synergy <1) | CI | CI | CI | CI | CI | CI |
| JVM3 | 0,69508 | 1,07658 | 1,03835 | 0,57964 | 0,35634 | 0,19225 |

**Figure S8**. Synergistic efficacy of AA and CPI-613, oligomycin A or metformin in OSU-CLL and JVM3 cell lines. A and B: left Panels: CellTiter-Glo assay was performed to detect cell kill synergy after 24 h in OSU-CLL and JVM3 cells treated with ascorbic acid (AA) and CPI-613, oligomycin A or metformin. The curves show the dose-effect of single drugs and of drugs combination. Each value is the mean of one experiment in duplicate. Right panels: Tables show the combination index (CI). The CI values were calculated using the Chou-Talalay method by the software Compusyn. Fa : fraction affected (fraction of cells affected by a particular drug dose).

| Patient | Sex | Age | CD38 | Matutes score | Karyotype | IGHV mutation | Response to AA |
|---|---|---|---|---|---|---|---|
| #1 | M | 62 | Negative | 5 | Normal | ND | Sensitive |
| #2 | F | 88 | Negative | 4 | ND | ND | Sensitive |
| #3 | F | 72 | Negative | 5 | Trisomy 12 / del13q | ND | Sensitive |
| #4 | F | 82 | ND | 5 | del 13q | ND | Resistant |
| #5 | M | 62 | ND | 5 | t(8;18) | ND | Resistant |
| #6 | M | 59 | Negative | 4 | ND | ND | Sensitive |
| #7 | F | 84 | Negative | 4 | ND | ND | Sensitive |
| #8 | M | 62 | Negative | 5 | ND | ND | Sensitive |
| #9 | M | 87 | Negative | 5 | Normal | ND | Sensitive |
| #10 | M | 53 | Negative | 5 | Normal | ND | Sensitive |
| #11 | F | 90 | Negative | 5 | ND | ND | Sensitive |
| #12 | M | 72 | Negative | 5 | Normal | ND | Sensitive |
| #13 | M | 65 | Negative | 5 | ND | ND | Sensitive |
| #14 | M | 72 | ND | ND | ND | | Sensitive |
| #15 | F | 61 | Negative | 5 | ND | ND | Resistant |
| #16 | F | 72 | Negative | 5 | ND | ND | Sensitive |
| #17 | F | 67 | Positive | 4 | Trisomy 12, 19 / del 13q | ND | Resistant |
| #18 | F | 74 | Negative | 5 | ND | ND | Sensitive |
| #19 | F | 85 | ND | 5 | ND | ND | Sensitive |
| #20 | M | 83 | ND | 5 | ND | ND | Sensitive |
| #21 | M | 70 | Negative | 5 | Anomaly of chr 22 | ND | Sensitive |
| #22 | F | 65 | Negative | 5 | ND | ND | Sensitive |
| #23 | M | 61 | ND | 5 | ND | ND | Sensitive |
| #24 | M | 63 | Negative | 5 | del 11q | Unmutated | Resistant |
| #25 | F | 63 | Negative | 5 | ND | ND | Sensitive |
| #26 | M | 60 | Negative | 5 | del13q | ND | Sensitive |
| #27 | M | 62 | Negative | 5 | del 13q | ND | Sensitive |
| #28 | F | 60 | Negative | 5 | Normal | ND | Sensitive |
| #29 | F | 76 | | | ND | | Sensitive |
| #30 | F | 63 | Negative | 5 | Normal | ND | Sensitive |
| #31 | M | 54 | Negative | 5 | Normal | ND | Resistant |
| #32 | M | 66 | ND | ND | ND | ND | Sensitive |
| #33 | M | 67 | ND | ND | ND | ND | Sensitive |
| #34 | F | 69 | ND | ND | ND | ND | Sensitive |
| #35 | M | 68 | Negative | 5 | ND | ND | Sensitive |
| #36 | M | 79 | Negative | 5 | ND | ND | Resistant |
| #37 | M | 60 | ND | ND | ND | ND | Sensitive |
| #38 | M | 60 | ND | ND | ND | ND | Sensitive |
| #39 | F | 54 | ND | ND | ND | ND | Sensitive |
| #40 | F | 94 | ND | ND | ND | ND | Sensitive |

**Table S1**. Features of the CLL patients included in the study. The CLL was staged according to Binet's classification. Matutes score was based on the expression of CD5, CD79, CD23, FMC7, and sIg. IGHV mutational status homology ≥ 98%: UM, unmutated; V3-21*, a subset conferring a worse prognosis. Cytogenetic abnormalities were determined by karyotyping and/or FISH. The CLL patient's karyotype was classified as follows: normal (no abnormalities detected), del (deletion), or ND (not determined). Response to ascorbic acid (AA) was determined by assessing cell viability after 24 hours treatment with AA (250 µM) (i.e. resistant: >50% cell viability after AA treatment).
